# Synthesis of doxorubicin-loaded peptosomes hybridized with gold nanorod for targeted drug delivery and CT imaging of metastatic breast cancer

**DOI:** 10.1186/s12951-022-01607-2

**Published:** 2022-08-31

**Authors:** Maliheh Hasannia, Khalil Abnous, Seyed Mohammad Taghdisi, Sirous Nekooei, Mohammad Ramezani, Mona Alibolandi

**Affiliations:** 1 Pharmaceutical Research Center, Pharmaceutical Technology Institute, Mashhad University of Medical Sciences, Mashhad, Iran; 2 Department of Pharmaceutical Nanotechnology, School of Pharmacy, Mashhad University of Medical Sciences, Mashhad, Iran; 3 Department of Medicinal Chemistry, School of Pharmacy, Mashhad University of Medical Sciences, Mashhad, Iran; 4 Targeted Drug Delivery Research Center, Pharmaceutical Technology Institute, Mashhad University of Medical Sciences, Mashhad, Iran; 5 Department of Radiology, Faculty of Medicine, Mashhad University of Medical Sciences, Mashhad, Iran; 6 Department of Pharmaceutical Biotechnology, School of Pharmacy, Mashhad University of Medical Sciences, Mashhad, Iran; 7 Student Research Committee, School of Pharmacy, Mashhad University of Medical Sciences, Mashhad, Iran; 8 Pharmaceutical Technology Institute, Nanotechnology Research Center, Mashhad University of Medical Sciences, Mashhad, Iran

**Keywords:** Peptosome, Doxorubicin, Gold nanorod, Breast cancer, Theranostics

## Abstract

**Background:**

Cancer nanomedicines based on synthetic polypeptides have attracted much attention due to their superior biocompatibility and biodegradability, stimuli responsive capability through secondary conformation change, adjustable functionalities for various cargos such as peptides, proteins, nucleic acids and small therapeutic molecules. Recently, a few nanoformulations based on polypeptides comprising NK105, NC6004, NK911, CT2103, have entered phase I-III clinical trials for advanced solid tumors therapy. In the current study, we prepared polypeptide-based vesicles called peptosome via self-assembly of amphiphilic polypeptide-based PEG-PBLG diblock copolymer.

**Results:**

In this regard, poly(γ-benzyl L-glutamate (PBLG) was synthesized via ring opening polymerization (ROP) of γ-benzyl L-glutamate-N-carboxyanhydride (BLG-NCA) using N-hexylamine as initiator. Then amine-terminated PBLG was covalently conjugated to heterofuctional maleimide PEG-carboxylic acid or methyl-PEG-carboxylic acid. The PEG-PBLG peptosomes were prepared through double emulsion method for the co-delivery of doxorubicin.HCl and gold nanorods as hydrophilic and hydrophobic agents in interior compartment and membrane of peptosomes, respectively (Pep@MUA.GNR-DOX) that DOX encapsulation efficiency and loading capacity were determined 42 ± 3.6 and 1.68 ± 3.6. Then, theranostic peptosomes were decorated with thiol-functionalized EpCAM aptamer throught thiol-maleimide reaction producing Apt-Pep@MUA.GNR-DOX for targeted delivery. The non-targeted and targeted peptosomes showed 165.5 ± 1.1 and 185 ± 4.7 nm diameters, respectively while providing sustained, controlled release of DOX. Furthermore, non-targeted and targeted peptosomes showed considerable serum stability. In vitro study on MCF-7 and 4T1 cells showed significantly higher cytotoxicity for Apt-Pep@MUA.GNR-DOX in comparison with Pep@MUA.GNR-DOX while both system did not show any difference in cytotoxicity against CHO cell line. Furthermore, Apt-Pep@MUA.GNR-DOX illustrated higher cellular uptake toward EpCAM-overexpressing 4T1 cells compared to Pep@MUA.GNR-DOX. In preclinical stage, therapeutic and diagnostic capability of the prepared Pep@MUA.GNR-DOX and Apt-Pep@MUA.GNR-DOX were investigated implementing subcutaneous 4T1 tumor model in BALB/c mice. The obtained data indicated highest therapeutic index for Apt-Pep@MUA.GNR-DOX compared to Pep@MUA.GNR-DOX and free DOX. Moreover, the prepared system showed capability of CT imaging of tumor tissue in 4T1 tumorized mice through tumor accumulation even 24 h post-administration.

**Conclusion:**

In this regard, the synthesized theranostic peptosomes offer innovative hybrid multipurpose platform for fighting against breast cancer.

**Graphical Abstract:**

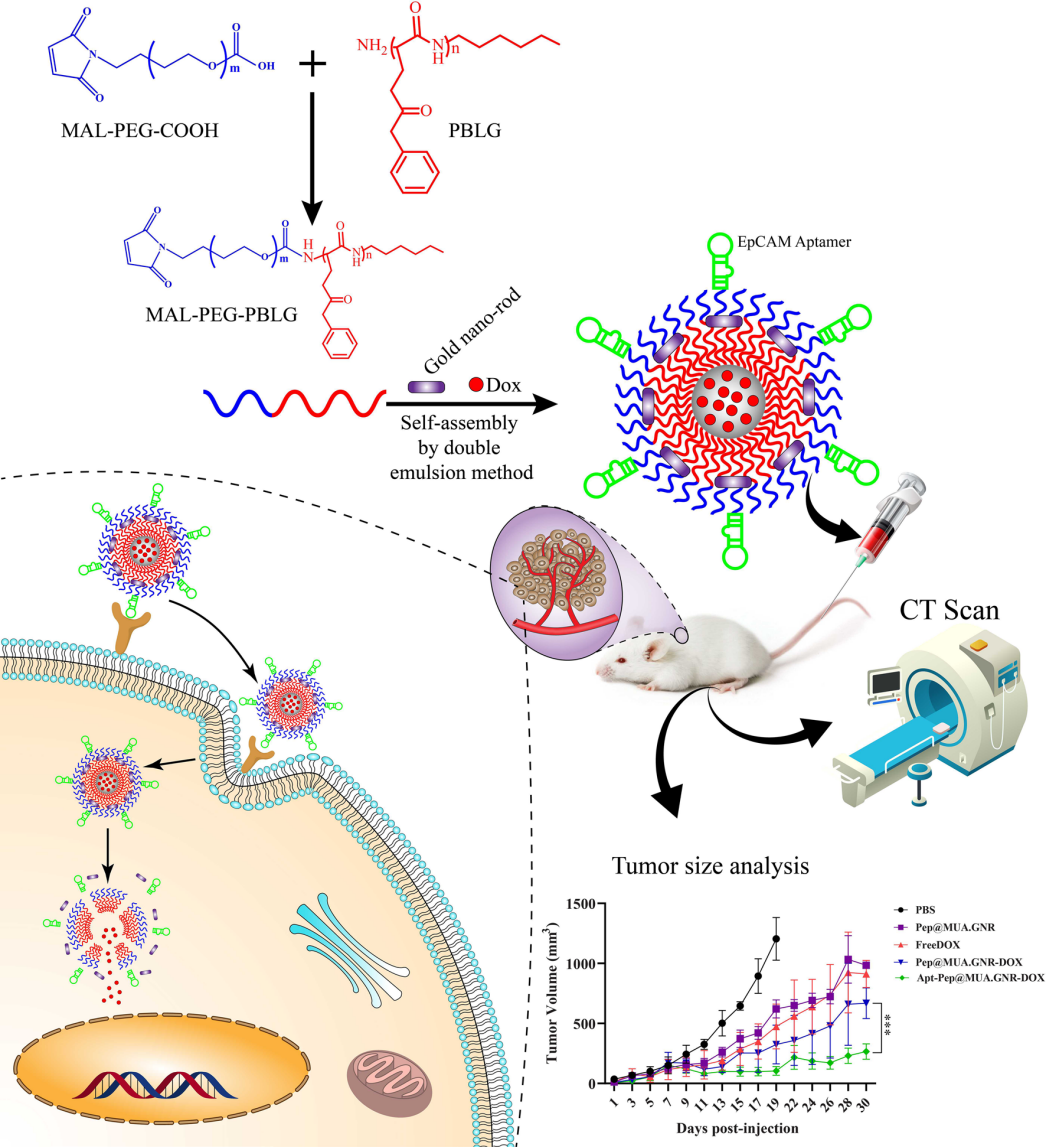

## Introduction

In recent years, more than 100 types of cancer were discovered among which breast cancer (BC) is one of the most popular diagnosed cancer in women. According to statistics, 12–14% of all identified cancer cases in the world are BC [1]. Moreover, 2.1 million women died in 2018 due to BC and every 18 s one BC patient is diagnosed [2]. In recent years, different therapies including surgery, radiation and diverse forms of chemotherapy have been implemented to control BC in various stages [3]. Doxorubicin (DOX) is a typical anthracycline antibiotic with high antitumor efficacy that has been applied to cure cancers such as osteosarcoma, lymphomas, esophageal carcinomas and breast cancer [4, 5]. However, DOX leads to severe side effects including myocardial dysfunctions and depression of bone marrow [6]. The aforementioned side effects could be reduced significantly by providing selective, guided delivery of chemotherapeutics to the site of action. The guided delivery of chemotherapeutics significantly increases their therapeutic index while reducing their systemic toxicity [7].

Extensive investigations demonstrated that BC in early stage (when cancer cells restricted to the axillary lymph nodes or breast) is usually curable by traditional chemotherapeutics but metastatic BC could not entirely be treated via conventional chemotherapeutics [8]. As a result, in recent years, design and development of targeted drug delivery platforms have been increased for the treatment of advanced or metastatic breast cancer [9].

Amphiphilic block copolymers could be self-assembled to various nano-objects including cylinders, nanofibers, micelles and vesicles, depending on architecture, chemical composition and molecular weights of the block copolymers [10]. Polypeptide-based block copolymers have indicated unique advantages compared to other synthesized amphiphilic block copolymers comprising excellent biodegradability and biocompatibility, well-defined secondary conformations including α-helix and β-sheet and adjustable functionalities for delivery of various payloads such as nucleic acids, proteins, peptides and synthetic drugs [11]. Kataoka et al. designed several micellar nanostructures through poly(ethylene glycol)-*b*-polypeptide block copolymers such as NK105, NC6004, NK911, etc. that have been used in clinical trials for treating different kinds of solid tumors [12]. Among different polypeptide-based block copolymers, poly(ethylene glycol)-b-poly(glutamic acid) have been widely investigated as peptomicelles (micellar nanostructure based on amphiphilic copoly peptides) in preclinical and clinical evaluations [13]. For instance, NC-4016, NK012 and NC-6004 have been under investigation in phase I, II and III clinical studies, respectively [14]. All of these structures are prodrugs of chemotherapeutics, NC-6004 is comprised of PEG-PGA polypeptide bound to cisplatin and self-assembled to peptomicelles with size of 30 nm [15]. The therapeutic peptomicelles, NC-6004 was evaluated in phase III clinical trial for treating pancreatic cancer and the obtained results demonstrated enhanced tumor penetration of this platform to the pancreas solid tumor with dense stroma compared to albumin (90 nm) or liposomal nanocarriers (130 nm) [16].

Recently, Ram et al*.* synthesized SN38-conjugated PHPMA-*b*-PGA and self-assembled it to a therapeutic peptomicelles. The synthesized structure showed great therapeutic index against colon adenocarcinoma in preclinical stage [17].

Among several strategies for the synthesis of polypeptides, ring-opening polymerization (ROP) of N-carboxyanhydrides (NCAs) is one of the versatile method for the synthesis of polypeptides applied for bio-imaging, tissue engineering, antimicrobials, drug and gene delivery [18]. In this regard, ROP of NCA was used to prepare polypeptides with unique properties such as narrow polydispersities, adjustable molecular weight, desirable side chain functionalities, great optical purity and large scale production [11, 19]. Progress in NCA chemistry has led to design different kind of platforms based on synthetic polypeptides such as hydrogels, hybrid nanoparticles (NPs), nucleic acid complexes, micelles and vesicles [20]. Recently, due to the encapsulation limitations of micellar structures, vesicular NPs have attracted more attention [21]. These vesicular systems named peptosomes, are formed via self-assembly of polypeptide-based amphiphilic block copolymers [22]. Peptosomes, similar to polymersomes, could simultaneously encapsulate hydrophilic and hydrophobic molecules in aqueous core and hydrophobic bilayer, respectively [23]. On the contrary, polypeptide-based micelles (peptomicelles) can only be loaded with hydrophobic molecules in their hydrophobic cores [24].

Similar to polymersomes, peptosomes have more advantages in comparison to liposomes including controlled membrane thickness with changing molecular weight of polypeptide-based amphiphiles, higher stability, and lower permeability [25] while providing great properties due to the presence of polypeptide blocks in their structures. In the last decade, development of theranostic nanoplatforms for simultaneous therapy and diagnosis of cancer is considered as a priority in this field [26]. These nano-platforms are capable of simultaneous co-encapsulation of diagnostic and therapeutic agents [27]. In this regards, vesicular systems such as pepetosomes, are one of the superior nanocarriers for theranostic applications due to their additional advantages including excellent loading capacity of hydrophobic and hydrophilic agents, and facile functionalization of the block chains for designing trigger responsive and targeted delivery systems [28].

Targeted theranostic nanocarriers have been applied to selectively accumulate anticancer and diagnostic agents in tumor site for increasing therapeutic index while providing opportunity for real time monitoring of tumor response to the therapeutic agent and evaluate its physiological nature [29]. Gold nanorods (GNRs) have superior advantages in comparison with spherical gold NPs including unique optical attributes due to potent surface plasmon resonance (SPR) properties, having two distinguished plasmon bands with adjustable longitudinal absorption band from visible to near-infrared (NIR) areas while spherical gold NPs have only single plasmon band in the visible area [30]. Furthermore, great stability and enduring high salt concentrations (above 0.5 M) are created by GNRs due to cetyltrimethylammonium bromide (CTAB) either adsorbed on the surface of GNRs or free in the solution for complex real sample tracing [31]. The physiochemical features of GNRs including shape, charge and surface functional groups influence on the amount of uptake, elimination and toxicity of GNRs [32]. As a result, these physiochemical characteristics increased the biomedical applications of GNRs. On the other hand, GNRs are widely implement in biomedical research due to its unique properties including strong extinction coefficient with sharp curve width, excellent photothermal conversion efficiency, high sensitivity to local dielectric constant shifts Near-Infrared Resonance (NIR) biomedical imaging [33], Optical Coherence Tomography (OCT) and X-ray computed tomography (X-ray CT) [34]. Thus, GNRs could be applied as diagnostic and treatment agents in a single platform. Additionally, other studies indicated that theranostics based on GNRs, have remarkable advantages compared to other theranostic systems such as the possibility of careful adjustment of time and place for all practical modalities [35].

GNRs as safe, non-toxic inorganic imaging agents, are suitable for computed tomography (CT) imaging due to higher X-ray absorption coefficient and atomic weight compared with iodine which has been widely applied as CT contrast agent [36]. Thus GNRs can be encapsulated in different carriers and used for bioimaging purposes in preclinical stage [21].

Decoration of different targeting ligands such as antibodies, aptamers, and folic acid on the theranostic nanoplatforms surface have improved the efficacy of both contrast agents and therapeutic drugs by targeted accumulation in the tumor site or the specific tissues. Recent reports demonstrated that the conjugation of NPs with aptamer improved the contrast agent capability due to desirable specificity and affinity for binding to tumor cells overexpressing a targeting ligand receptor [37–39].

The epithelial cell adhesion molecule (EpCAM) is a glycosylated protein in membrane of cell with 314 amino acids [40], that promotes cell growth through upregulation of the c-myc oncogene and cyclins A/E [41]. The transmembrane EpCAM protein can adjust various processes comprising regulation of cell proliferation, cell migration, Ca^2+^ independent cell–cell adhesion in epithelium and cell signaling [42]. Previous studies indicated that EpCAM was overexpressed up to 1000-fold in epithelial of cancerous tissues compared to healthy epithelial cells [43]. Due to the diverse expression level of EpCAM, this protein is appropriate candidate for targeted delivery of therapeutic and diagnostic agents for cancer therapy and diagnostic purposes. Previously, it was demonstrated that overexpression of EpCAM in breast cancer caused tumor relapses, metastatic progression and poor survival [44]. It was shown that progenitor or stem cells of breast, colorectal and pancreatic cancers are EpCAM positive [45].

Herein, we designed a peptosome nanocarrier based on polyethylene glycol-*block*-poly(γ-benzyl l-glutamate) (PEG-PBLG). In this regard, PBLG was synthesized through ROP of γ-benzyl-l-glutamate-N-carboxyanhydride (BLG-NCA) monomer using *n*-hexylamine as initiator. Then, PEG was covalently conjugated to PBLG via 1-ethyl-3-(3-(dimethylamino)propyl)carbodiimide HCl (EDC) and N-hydroxysuccinimide (NHS). This diblock copolymer can encapsulate hydrophobic GNR within the hydrophobic membrane and DOX into the aqueous interior of peptosome via double emulsion method. Cellular uptake and cytotoxic profile of peptosome nanoformulation was examined on MCF-7 and 4T1 cells as human and mouse breast cancer cell lines. Finally, theranostic capability of the nanopalatform was investigated on 4T1 bearing mice.

## Results and discussion

### Synthesis and characterization of PBLG

**Fig. 1:**
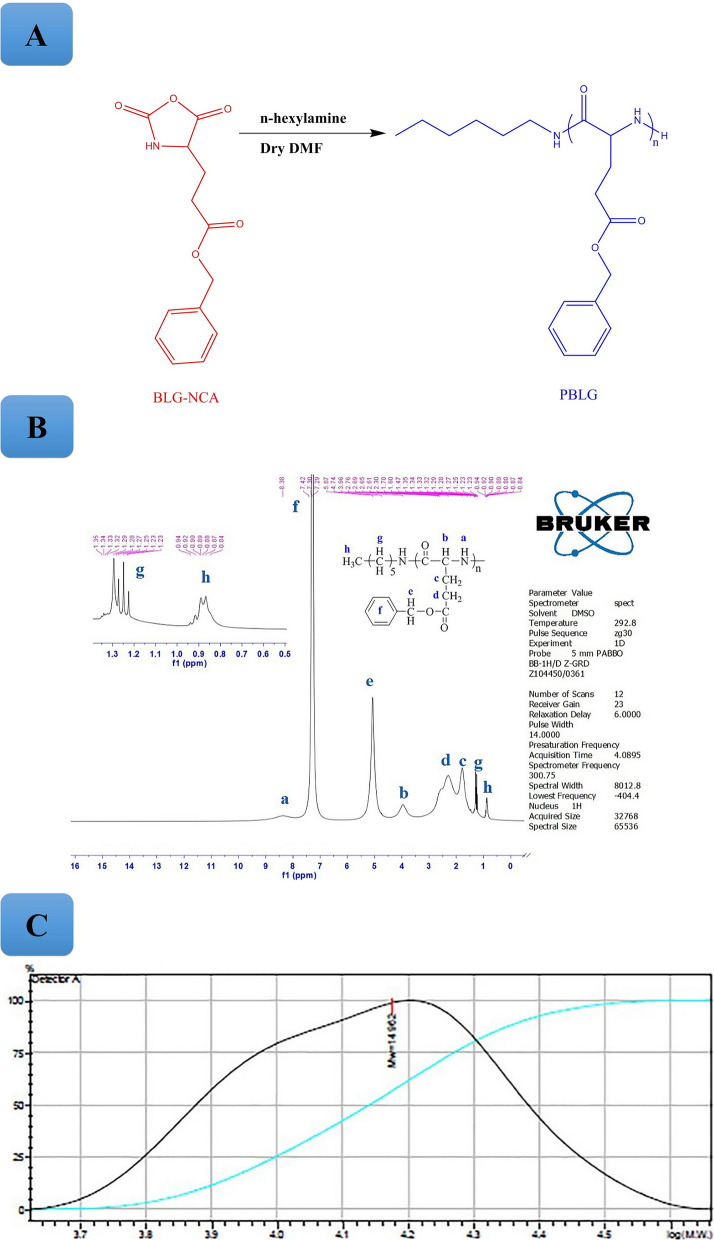
**A** ^1^HNMR spectrum of PBLG **B** GPC chromatogram of PBLG

**Fig. 2:**
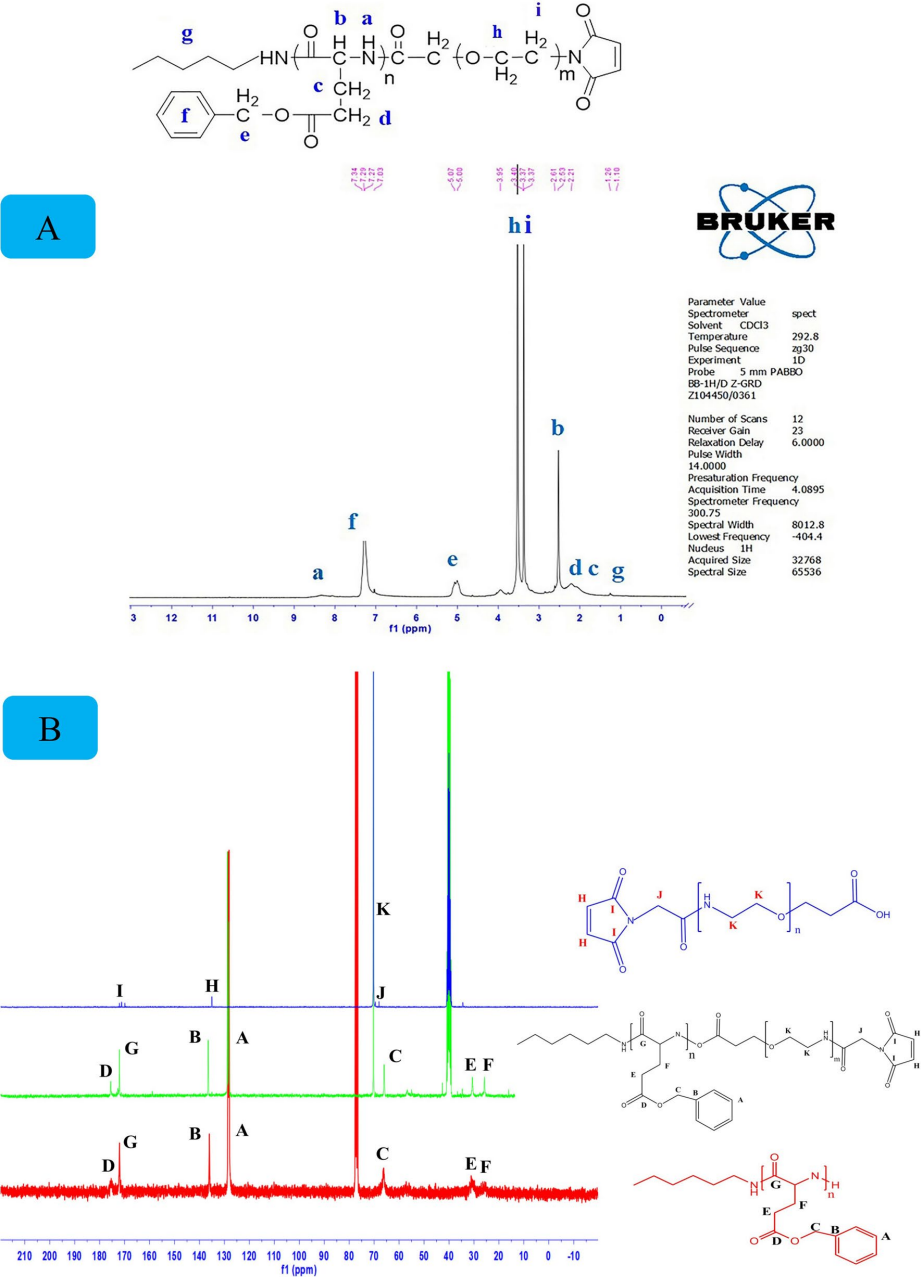
**A** ^1^HNMR spectrum of Mal-PEG-PBLG diblock copolymer. **B** ^13^CNMR spectrum of PBLG (red), Mal-PEG-COOH (blue), and Mal-PEG-PBLG block copolymer (green)

ROP of NCAs is the most desirable method to synthesize polypeptides. Polymerization under mild conditions using primary amines yielded narrow molecular weight distribution [23]. In this report, PBLG as hydrophobic block of amphiphilic block copolymer was successfully synthesized using the ROP of BLG-NCA through initiator (*n*-hexylamine) with monomer/initiator molar ratio of 120 [46]. The structural properties of the prepared PBLG was confirmed by ^1^HNMR. Figure 1 illustrated the ^1^HNMR spectrum of PBLG. The characteristic resonance signals of methylene groups (**–CH**_**2**_**CH**_**2**_COO) was observed at 1.60–1.78 ppm (**c**) and 1.80–2.76 ppm (**d**). Furthermore, peaks appeared at 3.96 ppm (**b**, –**CH–**), 5.07 ppm (**e**, O**CH**_**2**_Ph), 7.29–7.42 ppm (**f**, aromatic ring, OCH_2_**–Ph**), and 8.38 ppm (**a**, –**NH–**) were assigned to PBLG. Also, the characteristic signals corresponding to *n*-hexylamine segment was observed at 0.84–0.94 ppm (**h**, **–CH**_**3**_) and 1.23–1.35 ppm (**g**, –**(CH**_**2**_**)**_**5**_–). Additionally, chemical structure of PBLG was further confirmed through the FTIR spectrum. According to this spectrum (Fig. 2B), the stretching vibration absorption peaks at 3295 cm^−1^ (**CO–NH**), 2810–2951 cm^−1^ (**C–H** aliphatic), 3033–3063 cm^−1^ (**C–H** aromatic), 1736 cm^−1^ (**COOR**), 1654 cm^−1^ (**CONH**), 1547 cm^−1^ (bending **N–H** in amide) and the deformation vibration of benzene ring at 739 cm^−1^ and 697 cm^−1^) were attributed to PBLG. The molecular weight and molecular weight distribution of the synthesized PBLG were evaluated using GPC analysis (Fig. 1, Table 1). According to the GPC analysis, the synthesized PBLG showed unimodal chromatogram with molecular weight of 14962 Da and narrow molecular weight distribution of 1.19. Thus, it could be concluded that the ROP of NCAs produced precisely-controlled molecular weight of the polymer.

**Table 1:** Polymer characteristics determined by GPC

polymer	GPC results		
	M_n_^a^	M_w_^b^	M_w/_M_n_^c^
PBLG	12519	14962	1.19510

^a^Average molecular number

^b^Average molecular weight

^c^Polydispersity

### Synthesis and characterization of Mal-PEG-PBLG

**Fig. 3:**
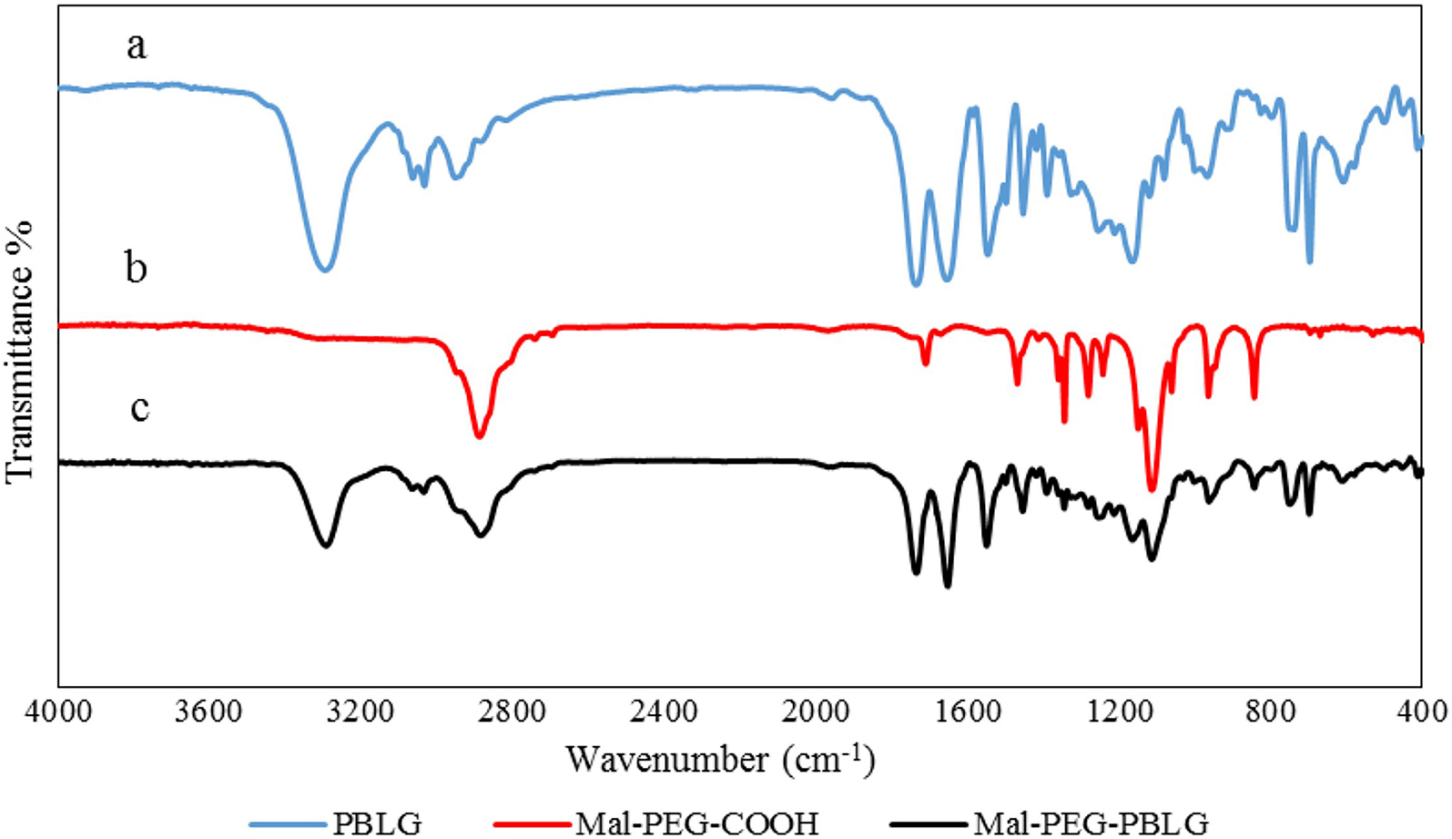
FTIR spectra of PBLG **a**, Mal-PEG-COOH **b**, and Mal-PEG-PBLG block copolymer **c**

The EDC/NHS amide conjugation reaction was applied for prepration of Mal–PEG-PBLG copolymer by coupling reaction between PBLG-NH_2_ and Mal-PEG-COOH. Chemical structure of Mal-PEG-PBLG wasconfirmed via ^1^HNMR, ^13^CNMRand FTIR spectrum Fig. 2A, B and C. The ^1^HNMR spectrum of Mal–PEG-PBLG indicated peaks corresponding to both PBLG and Mal-PEG-COOH blocks. The peak corresponding to CH_2_ of PEG block was observed at 3.37–3.5 ppm (peaks g and h), confirming the successful conjugation of PEG to PBLG. The successful coupling reaction between PBLG-NH_2_ and Mal-PEG-COOH was further confirmed by ^13^CNMR (Fig. 2B). In this regard, the peak corresponding to PEG (CH_2_-CH_2_-O) appeared at 71 ppm (peak K) and peaks corresponding to PBLG were present at 25 ppm (E, **C**H_2_COO), 32 ppm (F,**C**H_2_-CH-CO), 65 ppm (C, **C**H_2_-Ph), 130 ppm (A, **Ph**), 136 ppm (B, **Ph**-CH_2_O), 171 ppm (G, **C**OCH) and 175 ppm (D, **C**OOCH_2_Ph). Furthermore, conjugation of Mal-PEG-COOH to PBLG-NH_2_ was confirmed by FTIR analysis. It shows the amide bond peak at 1651 cm^−1^ (NH-C = O) corresponding to Mal–PEG-PBLG copolymer and the disappearance of carbonyl group of carboxylic acid in PEG chain (1709 cm^−1^). In addition, peaks corresponding to both PEG and PBLG appeared in Mal–PEG-PBLG, which are shown in Fig. 3.

**Fig. 4:**
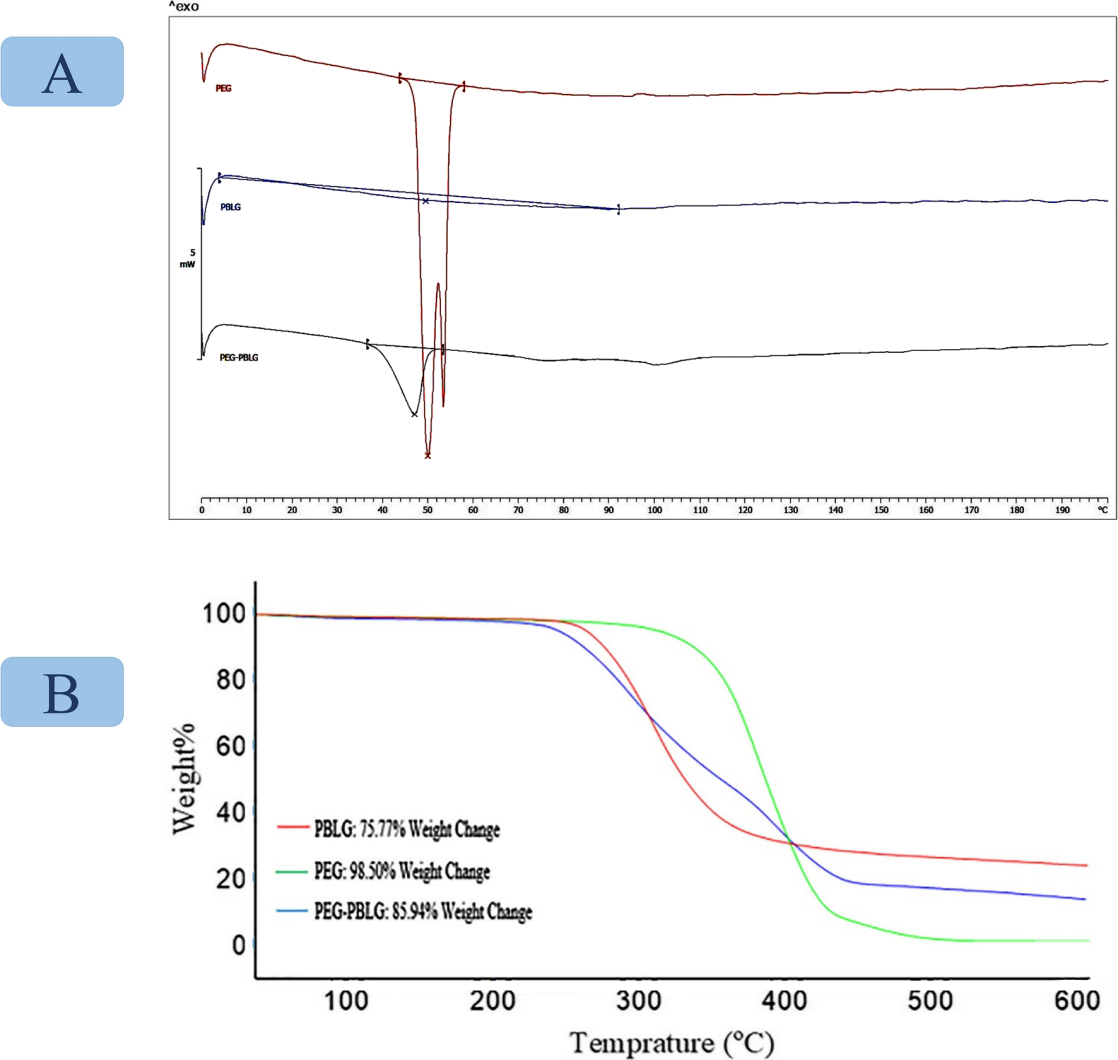
**A** Differential Scanning Calorimeter (DSC) of PEG (red), PBLG (blue), and PEG-PBLG (black), **B** TGA of PEG (green), PBLG (red), PEG-PBLG (blue)

The differential scanning calorimeter (DSC) thermogram of the PBLG indicated a broad peak at ~ 44–114 °C, while a single peak at 40–50 °C was observed in the thermogram of Mal-PEG-PBLG. According to this analysis, successful covalent conjugation of PEG to PBLG was further confirmed due to changing the melting endothermic bond to lower temperature (Fig. 4). The thermal stability of the diblock copolymer, hydrophilic and hydrophobic blocks, was evaluated by TGA (Fig. 4B). According to the TGA profile, the synthesized PBLG block and commercial Mal-PEG-COOH showed 75.77% and 98.50% weight loss, respectively by raising the temperature to 600 ºC. It should be noted that the weight loss of Mal-PEG-COOH started from higher temperature (350 ºC) with fast weight loss pattern while for PBLG block, the weight loss started from lower temperature (280 ºC) with slow weight loss pattern.

It is worth mentioning that the weight loss pattern for PEG-PBLG block was between PEG and PBLG ones which started from lower temperature in comparison with PEG while demonstrating higher weight loss pattern in comparison with PBLG block (Fig. 4).

### Optical and structural characterization of GNR and MUA-GNR

In recent years, GNRs have been widely studied as diagnostic probe in theranostic system due to several excellent properties including anisotropic optical and physicochemical properties, facile synthesis and possible surface modification for targeting and optical activation, high absorption ability at low amounts of GNR because of strong surface plasma resonance (SPR), easily adjustable longitudinal plasmon wavelengths in visible to NIR region via changing the aspect ratio of GNRs, great chemical stability and low cytotoxicity [47]. Among GNRs with various sizes, the nanoscale GNRs have been widely used for biomedical applications due to their unique properties including excellent dispersion ability, adjustable LSPR band in the NIR region, lower toxicity and faster clearance in vivo [48]. The seed-mediated method and the seedless method are more common approaches for the synthesis of small GNR. In this regard, seedless method has more advantages comprising (1) simple synthesis of GNR in large scale and good quality; (2) superior reproducibility; and (3) adjustable width as small as 8 nm [49]. Due to the advantages of small GNR for biomedical applications and their unique optical properties, in this study, seedless method was utilized to prepare small GNR [50]. Surfactants used in the process of the GNRs synthesis (CTAB) have limited their biological applications due to their high cytotoxicity. Thus, replacement of CTAB with thiol-terminated molecules via ligand exchange method is one the effective strategy for the surface modification of small GNRs. In this regard, we used 11-mercaptoundecanoic acid (MUA) as hydrophobic thiolated ligand to functionalize small GNRs to reduce its toxicity and encapsulate it in bilayer of peptosome. The successful replacement of CTAB with organic ligand was confirmed by zeta potential and FTIR spectroscopy [51].

**Fig. 5:**
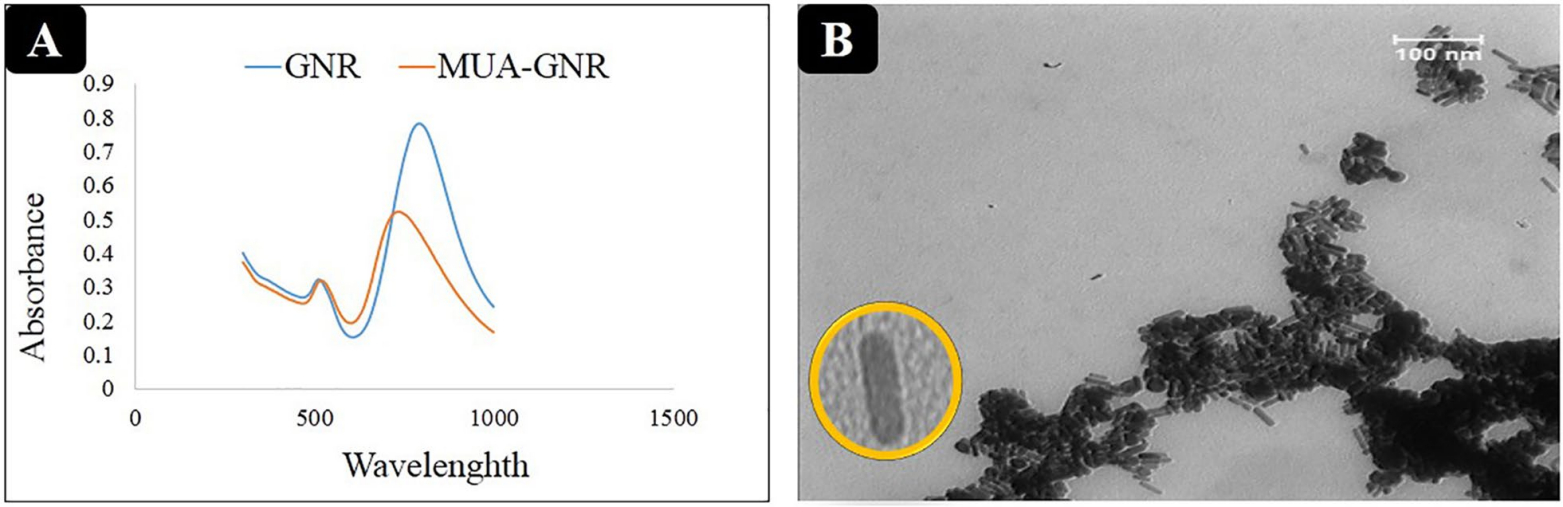
**A** UV spectra of GNR and MUA-GNR, **B** TEM image of MUA-GNR

**Fig. 6:**
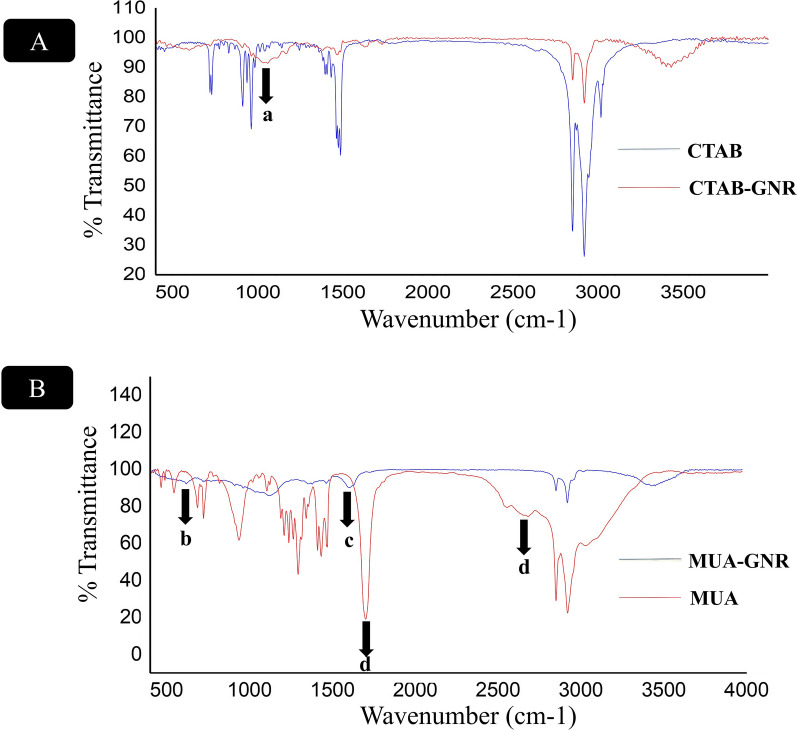
FTIR spectra of **A** CTAB (red) and CTAB-GNR (blue). FTIR spectra of **B** MUA (blue) and MUA-GNR (red)

The UV–visible absorption spectra of small GNRs and hydrophobic GNRs were analyzed with quartz cuvettes with 1 cm optical path length. The absorption spectra of GNR, MUA-GNR (GNR capped MUA) and the TEM image of MUA-GNR were represented in Figs. 5A, 6. According to UV spectra, the transverse plasmon wavelength (TPW) and the longitudinal plasmon wavelength (LPW) of GNRs appeared at about 794 and 512 nm, respectively*.* The replacement of CTAB by MUA was confirmed by measuring the zeta potential of the GNR. The zeta potential analysis indicated successful ligand exchange process due to the reduction of GNR surface charge from 21.8 ± 1.4 to −16.7 ± 0.4 mV after ligand replacement of positively charged CTAB with negatively charged MUA, verifying that most of CTAB were replaced by MUA [52–54].

Moreover, chemical structure of ligands on the surface of GNR was investigated by FTIR spectroscopy before and after the ligand exchange process. In this regard, FTIR spectrum of CTAB-capped GNR (Fig. 6A, red) indicated a peak at 1058 cm^−1^ (arrow, a) corresponding to stretching frequency of the quaternary amine of CTAB layer on the GNR surface. Besides, COOH stretch band (1699 cm^−1^, arrow d) appeared in FTIR spectrum of MUA (Fig. 6B, red) which was shifted to 1601 cm^−1^ (arrow c) in GNR-MUA spectrum (Fig. 4B, blue) due to the deprotonation of COOH groups. Existence of bands corresponding to C-S stretch (718 cm^−1^, arrow b) and elimination of S–H stretch (2682 cm^−1^, arrow d) in MUA-GNR demonstrated the successful ligand exchange [55–57].

In the other hands, the spectrum of MUA-GNR indicated the red shift in the longitudinal surface Plasmon peak due to its surface modification. During the ligand exchange, thiols bind to gold through Au–S bonds, which decrease the density of free electrons in the small GNR. Enhancing electron density lead to enhance the SPR frequency; accordingly, the SPR would be red-shifted (towards lower frequency) when the electron density was reduced [55].

The TEM image indicated rod morphology of the synthesized GNR with average diameter of 25 nm and desirable homogeneity (Fig. 5B).

### Preparation and characterization of peptosomes

Previous studies demonstrated that the hydrophilic volume fraction (*f*_*EO*_) of linear amphiphilic copolymers affected the morphology of the self-assembled NPs. Vesicular NPs could be formed when the *f*_*EO*_ of the amphiphilic copolymers are in the range of 25–40% [56–59]. For the first time, we successfully prepared peptosome based on PEG-PBLG with favorable *f*_*EO*_ (25%) through adjusting the feed ratio of BLG-NCA to *n*-hexylamine in ROP process. Single emulsion method was utilized for the formation of blank and MUA-GNR-loaded peptosomes. The internal core and membrane of peptosomes were loaded with hydrophilic DOX and hydrophobic MUA-GNR respectively via double emulsion method.

Encapsulation efficiency (EE) and loading content (LC) of DOX in peptosomes were calculated to be 42 ± 3.6 and 1.68 ± 3.6, respectively. On the other hand, the amount of MUA-GNR (Au content) encapsulated in peptosomes measured by inductively coupled plasma/optical electron microscopy (ICP-OES) was 0.33 wt%.

Size and polydispersity of the prepared peptosomes were determined through DLS and the results are represented in Table 2.

**Table 2:** Size and polydispersity index of the blank and co-encapsulated formulations

Composition	Size (nm) Z-Average	PDI	Zeta potential (mV)
Blank peptosome	149 ± 0.723	0.086	−14.1
Pep@MUA.GNR	151.3 ± 3.21	0.096	−14
Pep@DOX	147.8 ± 6.36	0.219	−9.55
Pep@MUA.GNR-DOX	165.5 ± 1.153	0.091	−5.35
Apt-Pep@MUA.GNR-DOX	185 ± 4.7	0.208	−1.69

The size of nanoparticulate systems influence both their blood circulation time and tumor accumulation [60]. It should be noted that NPs smaller than 200 nm significantly accumulate at tumor site due to their passive targeting capability after intravenous administration based on EPR effect (enhanced permeation and retention effect).In the current study, both nanoplatforms (Apt-Pep@MUA.GNR-DOX and Pep@MUA.GNR-DOX) showed appropriate size (smaller than 200 nm) with appropriate PDI for intravenous administration as cancer therapeutics [14, 44, 45].

Acumulation of the nanoparticulate systems in tumor microenvironment via EPR effect increases their therapeutic index while reducing their systemic toxicity.

Recent developments indicated that theranostic nanoplatforms based on biocompatible polymeric vesicles have exhibited ideal efficacy in terms of treatment and diagnosis [28].

Until now, various contrast agents were encapsulated in polymeric vesicles amongst which, small GNR showed desirable effectiveness and safety profile due to the high X-ray attenuation coefficient while clearing from the body through renal clearance [48, 58–60].

One of the important factor for safety of GNR is their capping agent. In this regard, GNRs with toxic CTAB capping are not suitable for biomedical applications. Therefore, extraction of CTAB was performed by ligand exchange process with thiol-terminated molecules due to strong AU–S conjugation [66].

According to the advantages of small GNRs as CT scan contrast agent and polymeric vesicles as promising vehicle, designing theranostic nanoplatforms based on vesicular structures and small GNR could provide theranostic capability with desirable safety toward developing cancer theranostic platforms.

In a study, DiazDuarte-Rodriguez et al. fabricated pH-responsive polymersomes based on poly(ethylene glycol)-b-poly(N,N-diethylaminoethyl methacrylate) (PEG-b-PDEAEM) [67]. This polymerome was simultaneously loaded with hydrophilic GNR and DOX but biological application of this system was not evaluated in vivo. It should be noted that the encapsulated GNR in this study was capped with toxic CTAB layer.

In the current study, for the first time, a hydrophobic small GNR with biocompatible non-toxic capping was co-encapsulated with DOX in polymeric vesicles based on PEG-PBGL. The fabricated innovative multimodal theranostic nanoplatform was extensively investigated in vitro and in vivo in terms of its biomedical potency.

### In vitro DOX release patterns

**Fig. 7:**
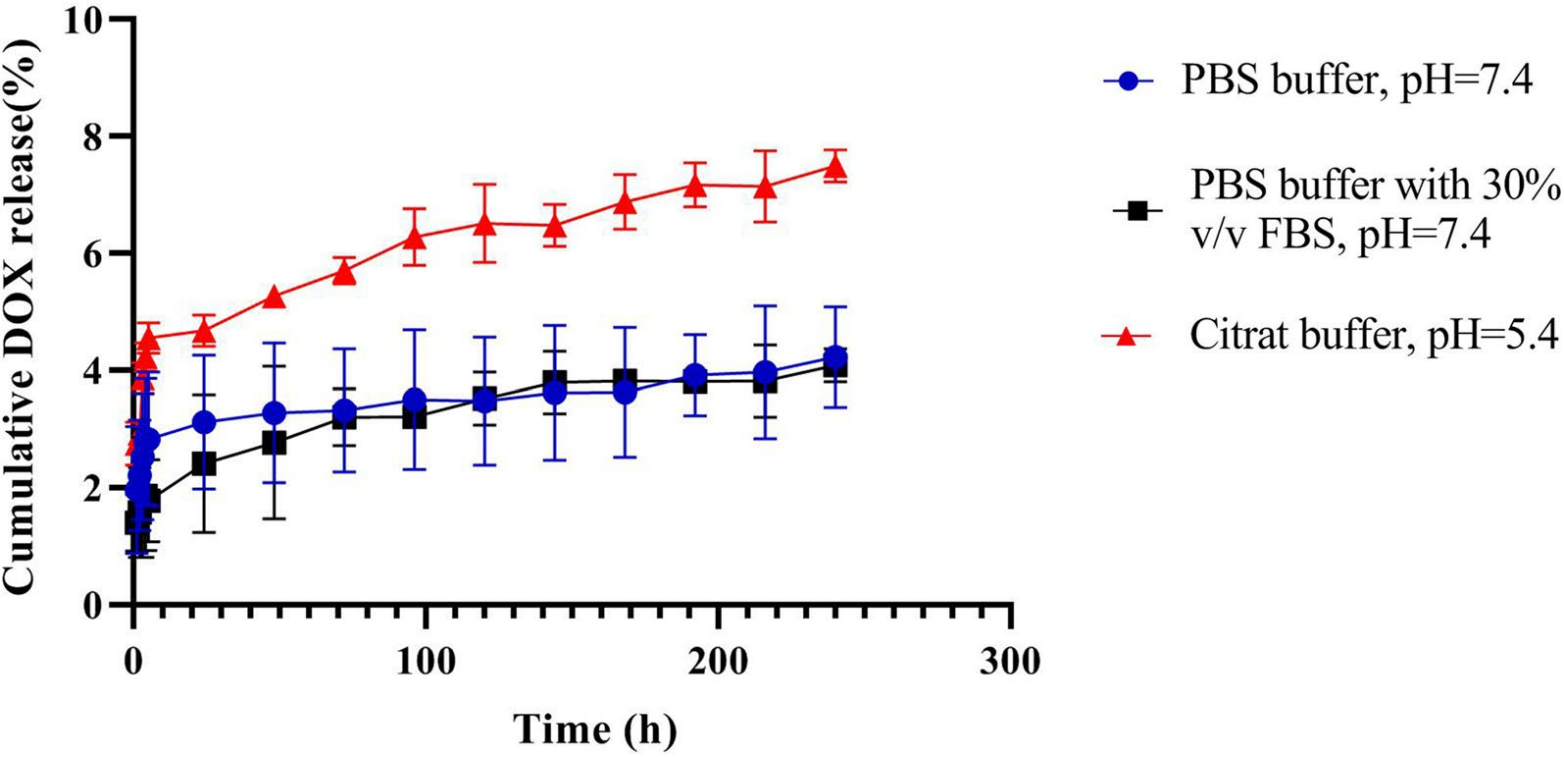
Release patterns of DOX from Pep@MUA.GNR-DOX in various release media including PBS (pH = 7.4), PBS supplemented with 30% v/v FBS, and citrate buffer (pH = 5.4). (Error bars show the standard error of mean for two different experiments in the same conditions).

In this regard, the release of therapeutic payload (DOX) from Pep@MUA.GNR-DOX in different release media ((phosphate buffered salin) PBS, PBS with 30% v/v FBS and citrate buffer) was investigated (Fig. 7). The results demonstrated that the amounts of DOX released from Pep@MUA.GNR-DOX in different buffer media was negligible. This might be due to the high stability PEG-PBLG block copoly peptide at different pH and under physiological conditions.

### Serum stability of peptosomes

**Fig. 8:**
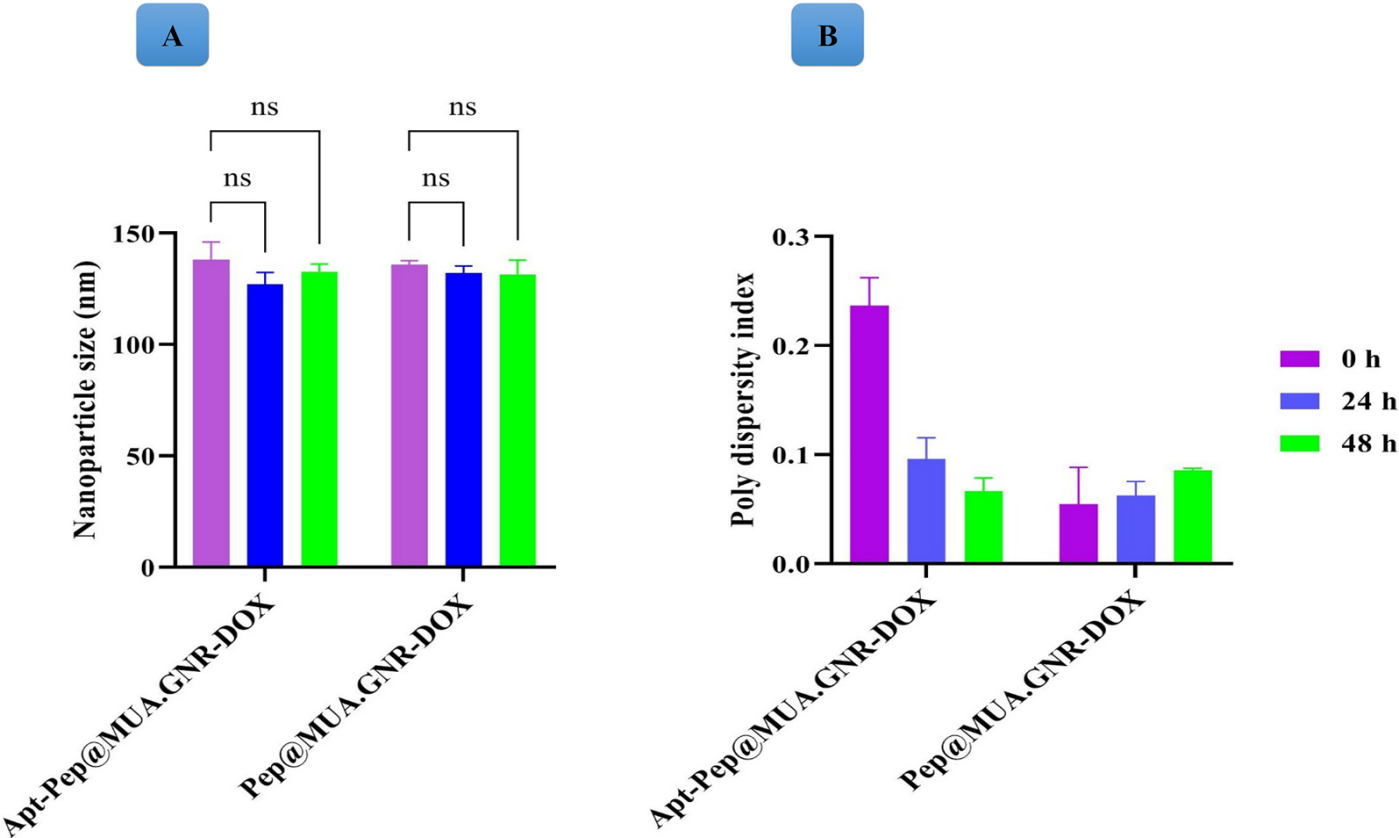
Serum stability of the Pep@MUA.GNR-DOX and Apt-Pep@MUA.GNR-DOX in PBS supplemented with 10% FBS after 0, 1 and 2 days incubation in a shaker incubator at 37 °C in terms of nanoparticle size (**A**) and polydispersity index

The effect of serum proteins on the size and polydispersity index of the targeted and non-targeted peptosomes were evaluated through DLS method (Fig. 8). The result of this study demonstrated excellent stability of the prepared peptosomes with narrow size dispersion in biological conditions during 48 h incubation. The observed uniform particle size dispersion of the fabricated peptosomes indicated the crucial role of PEGylation and aptamer decoration on the surface of this platform, which prevent protein adsorption and aggregation. In addition, shelf life of targeted and non-targeted peptosomes were evaluated after 30 days storage at 4 °C. According to this experiment, no change in particle size and PDI of the prepared peptosomes over 30 days storage at 4° C demonstrated desirable stability and shelf life of these systems.

### EpCAM aptamer conjugation

The prepared Pep@MUA.GNR-DOX was conjugated to thiol-modified EpCAM DNA aptamer via thiol-maleimide reaction. The maleimide functional group of PEG could be covalently linked to thiol end-terminal of EpCAM aptamer to prepare targeted peptosome (Apt-Pep@MUA.GNR-DOX). Aptamer conjugation on outer surface of Pep@MUA.GNR-DOX caused 20 nm size increment of Apt-Pep@MUA.GNR-DOX in comparison with Pep@MUA.GNR-DOX by DLS measurement. The size increment was previously reported after targeting ligand decoration on the surface of NPs [61, 68–74].

The yield of aptamer conjugation was indirectly calculated via measuring the absorption of washing solution of targeted nanoformulation at 260 nm that indicated 100% of aptamer was decorated on the peptosomes surface.

### Morphological investigation of targeted and nontargeted peptosomes

**Fig. 9:**
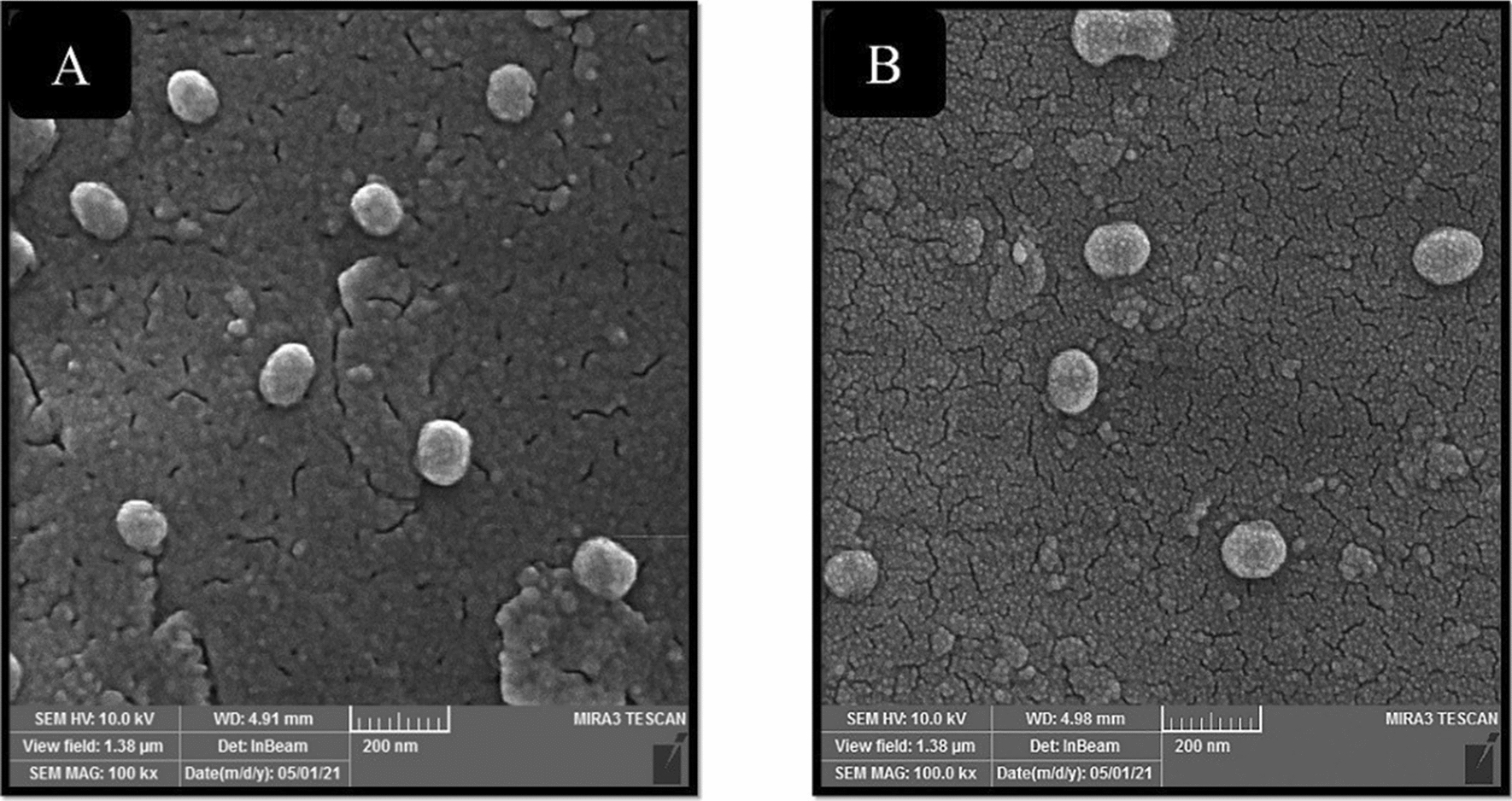
FE-SEM images of Pep@MUA.GNR-DOX (**A**) and Apt-Pep@MUA.GNR-DOX (**B**)

**Fig. 10:**
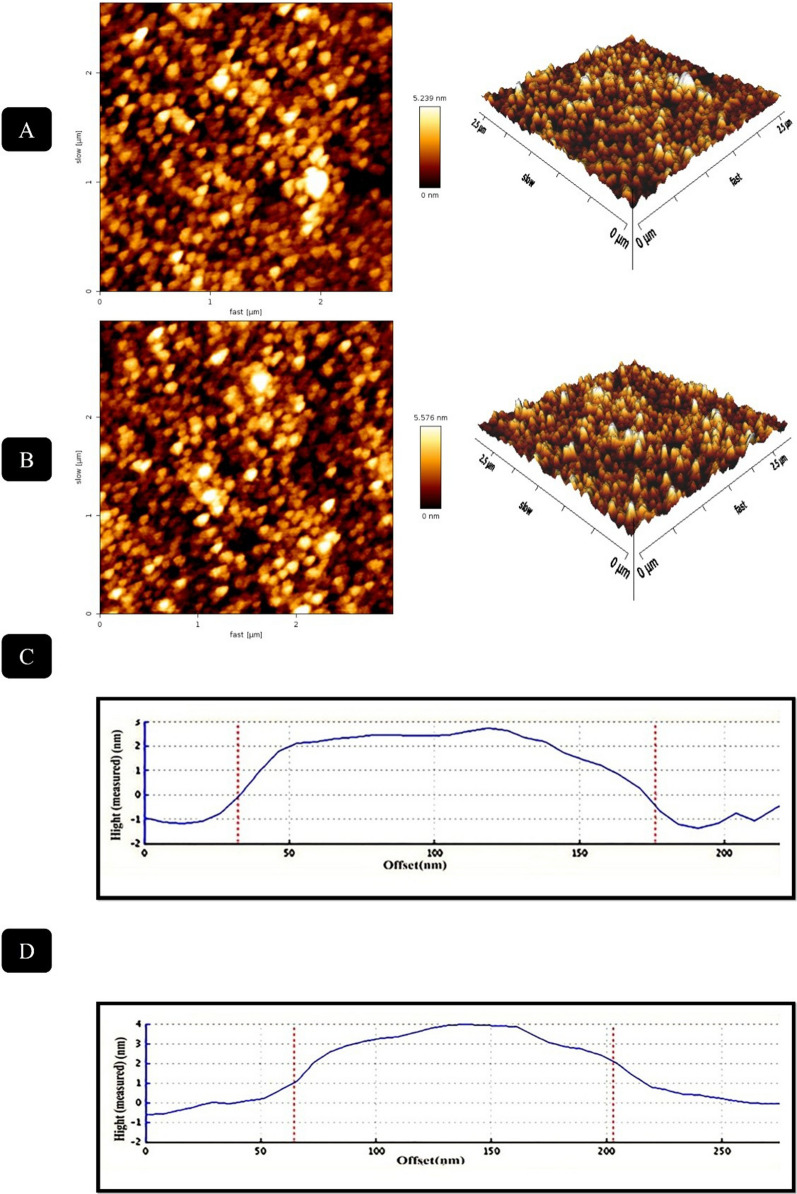
AFM analysis of Pep@MUA.GNR-DOX (**A**); Apt-Pep@MUA.GNR-DOX (**B**), Height profile of Pep@MUA.GNR-DOX (**C**) and Apt-Pep@MUA.GNR-DOX (**D**)

The morphological properties and size polydispersity of Apt-Pep@MUA.GNR-DOX and Pep@MUA.GNR-DOX were investigated via FE-SEM and AFM images (Figs. 9 and 10). The systems indicated spherical morphology and appropriate nanoscale size in FE-SEM images (Fig. 9) which is similar to the DLS results. Furthermore, more information about morphology and homogeneity of the targeted and non-targeted platforms were provided through AFM images (Fig. 10). The AFM analysis of peptosomes confirmed the spherical structure of nano-formulations with narrow size distribution.

### Cellular uptake

**Fig. 11:**
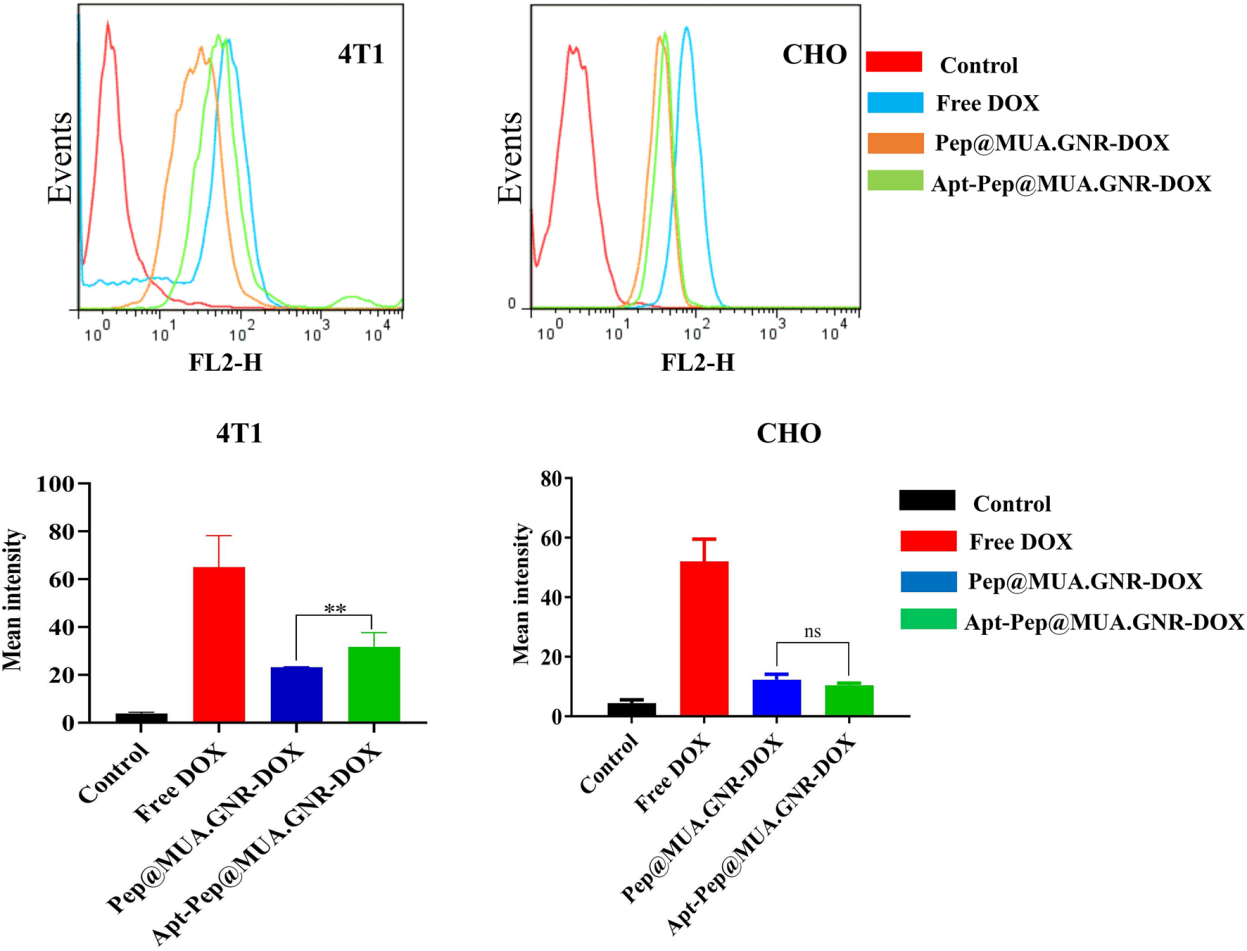
Flow cytometry analysis of CHO, and 4T1 cell lines for DOX cellular internalization evaluation after 2 h of exposure with either free DOX, Apt-Pep@MUA.GNR-DOX or Pep@MUA.GNR-DOX

Targeted platforms have been widely applied for specific delivery of nanoformulation to cancer cells. In the current study, the cellular internalization capability of the prepared systems, Pep@MUA.GNR-DOX, Apt-Pep@MUA.GNR-DOX and free DOX was evaluated using flow cytomerty analysis. For this purpose, the cellular uptake potential of Apt-Pep@MUA.GNR-DOX, Pep@MUA.GNR-DOX and free DOX in 4T1 and CHO cell lines (as EpCAM positive and negative cell lines respectively) was examined. According to the flow cytometry results illustrated in Fig. 11, greater cellular DOX internalization was observed in the targeted peptosomes compared to non-targeted ones in the 4T1 cell lines while cellular internalization of both targeted and non-targeted peptosomes was identical in CHO cells as EpCAM-negative cells. This data suggested a receptor-mediated endocytosis mechanism for EpCAM aptamer-targeted peptosomes in EpCAM overexpressed cells, 4T1 cell line. The EpCAM DNA aptamer used in this study, was capable of delivering different platforms selectively to EpCAM overexpressing cancer cells such as 4T1, MCF-7, C26 and HT29 [42, 43].

### In vitro cytotoxicity

**Fig. 12:**
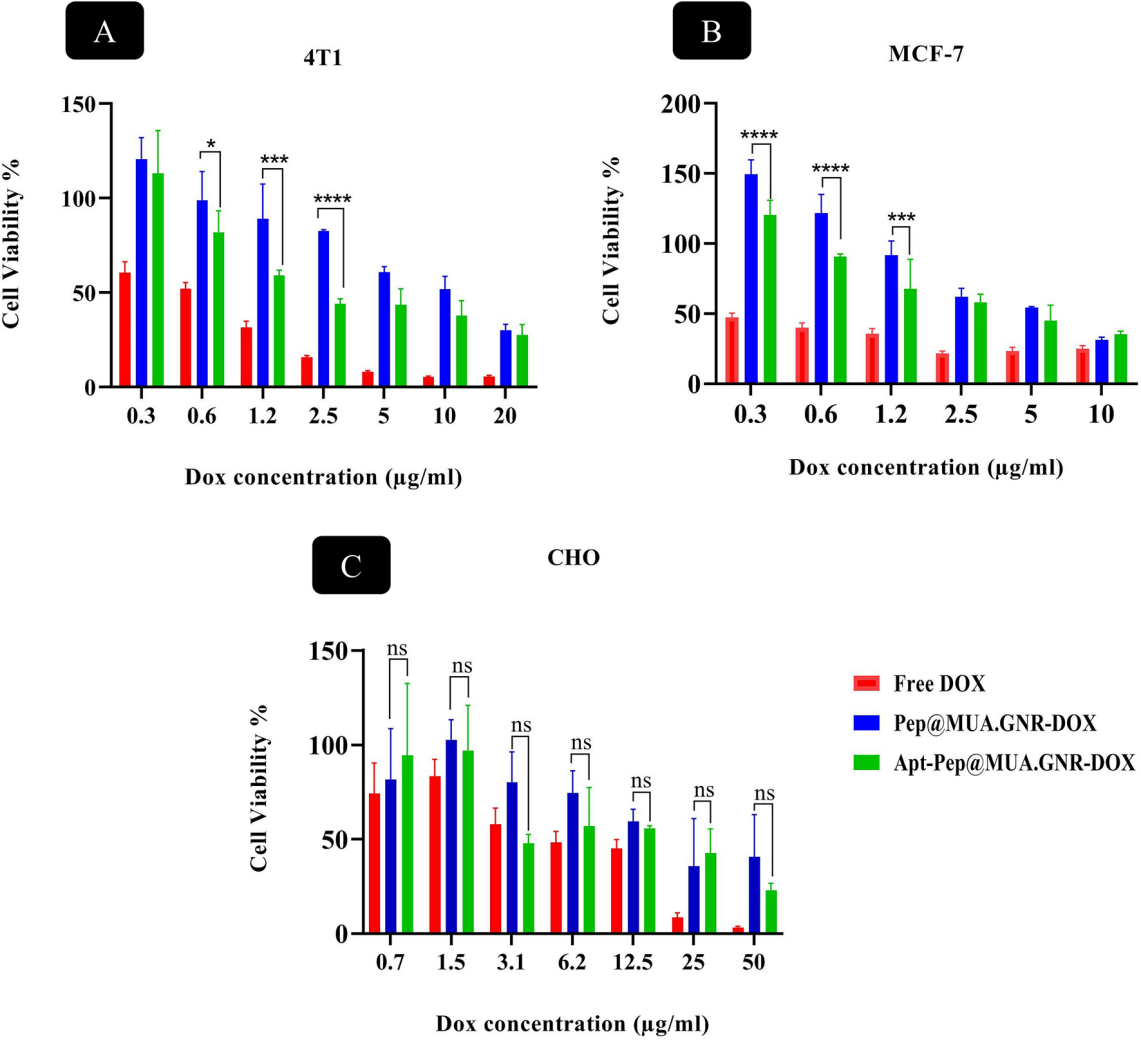
MTT assay of 4T1 (**A**), MCF-7 (**B**) and CHO (**C**) cell lines after 48 h exposure to free DOX, Apt-Pep@MUA.GNR-DOX, Pep@MUA.GNR-DOX at 37 °C

The in vitro cytotoxicity of free DOX, Apt-Pep@MUA.GNR-DOX and Pep@MUA.GNR-DOX was investigated in two overexpressed EpCAM cell lines (4T1 and MCF-7) and an EpCAM negative cell line (CHO) with the equal DOX concentrations ranging from 0.3 to 20 μg/ml (Fig. 12). Obtained results demonstrated significantly higher cytotoxicity for Apt-Pep@MUA.GNR-DOX compared to Pep@MUA.GNR-DOX in EpCAM-positive cells. Similar to uptake study, no obvious difference was observed between Apt-Pep@MUA.GNR-DOX and Pep@MUA.GNR-DOX in EpCAM-negative cell line (CHO). The cytotoxicity results on both 4T1 and CHO cells are in consistent with those obtained from flow cytometry analysis and showed that the difference in cytotoxicity is proportional to the extent of cellular uptake of targeted and non-targeted systems in EpCAM overexpressing cells. Previously, it was demonstrated that EpCAM acted as an effective targeting ligand for selective delivery of chemotherapeutics or imaging probes to cancerous cells due to EpCAM overexpression in primary and metastatic breast cancers [75–79].

It should be noted that EpCAM protein was overexpressed in most of human epithelial carcinomas, such as hepatic, colorectal, head and neck, breast and prostate cancers and specifically related to poor prognosis of breast cancer [55, 80].

In our study, free DOX showed higher cellular internalization and subsequently higher cellular toxicity in comparison with both targeted and non-targeted systems. The higher uptake and cytotoxicity of free DOX is might be due to abundant internalization of small molecule of DOX through cell membranes.

### In vivo antitumor activity and systemic toxicity

The in vivo therapeutic capability of Pep@MUA.GNR-DOX and Apt-Pep@MUA.GNR-DOX were compared to free DOX after single dose intravenous (i.v) administration of Pep@MUA.GNR, Pep@MUA.GNR-DOX, Apt-Pep@MUA.GNR-DOX and free DOX with equal DOX concentration (5 mg/kg) and MUA.GNR concentration (1 mg/kg) in 4T1 tumorized BALB/c mice.

**Fig. 13:**
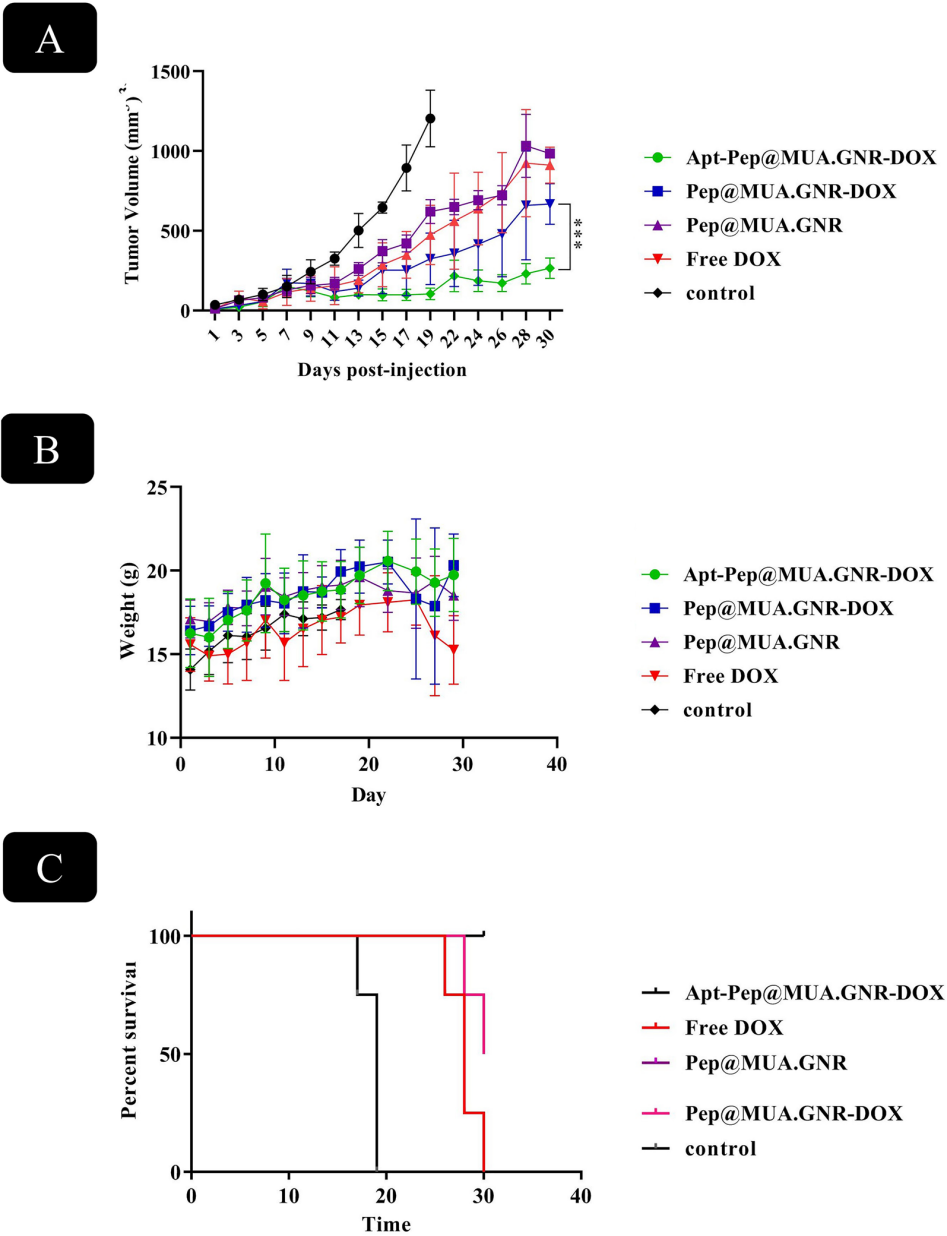
In vivo therapeutic efficacy study of Pep@MUA.GNR, Apt-Pep@MUA.GNR-DOX, Pep@MUA.GNR-DOX and free DOX with identical DOX (5 mg/kg) and MUA.GNR (1 mg/kg) concentration and PBS during 30 days after single dose i.v administration in 4T1 tumorized BALB/c mice. Tumor volume (**A**); Survival percentage (**B**) and Body weight (g) (**C**)

For this purpose, tumor volume, body weight loss and survival rate of the mice received either Pep@MUA.GNR, Pep@MUA.GNR-DOX, Apt-Pep@MUA.GNR-DOX, free DOX or PBS as negative control were followed for 30 days post-administration (Fig. 13). According to the obtained results, mice receiving either Apt-Pep@MUA.GNR-DOX or Pep@MUA.GNR-DOX showed enhanced tumor suppression in comparison with those receiving either Pep@MUA.GNR, free DOX or PBS. This could be ascribed to the capability of the noparticulate platform in passive targeting and tumor accumulation due to the EPR effect.

However, inhibition of tumor growth of mice receiving Apt-Pep@MUA.GNR-DOX was considerably higher in comparison with marginal tumor suppression in mice treated with Pep@MUA.GNR-DOX. The observed high tumor suppression efficacy in EpCAM aptamer-bioconjugated peptosomes, was due to the high binding affinity of the targeted system, Apt-Pep@MUA.GNR-DOX to EpCAM marker on the 4T1 surface and its consequent higher accumulation at the tumor site. As a result, binding of the targeted peptosomes to EpCAM receptors on the cancerous cells surfaces, led to the improved cytotoxicity and therapeutic efficacy of the Apt-Pep@MUA.GNR-DOX in comparison with that of Apt-Pep@MUA.GNR-DOX which could be attributed to the enhanced retention time of the Apt-Pep@MUA.GNR-DOX at the tumor microenvironment, thereby increasing cellular internalization of DOX and retarding the tumor extravasation of the targeted peptosomes.

Free DOX as a small hydrophilic molecule, circulates throughout the body post-administration and is vastly cleared from the blood circulation due to the renal clearance [81, 82]. Thus mice receiving free DOX did not indicate tumor growth inhibition compared to that of control group.

The body weight of mice and survival rate as indicators of systemic toxicity were represented in Fig. 13B, C. In this report, four out of five mice treated with Apt-Pep@MUA.GNR-DOX and two out of five mice treated with Pep@MUA.GNR-DOX were alive after 30 days i.v injection.However, all animals receiving free DOX died during 30 days of experiment. Moreover, four out of five mice receiving PBS died during 30 days, post-administration.

In a parallel experiment, the body weight of mice receiving either Pep@MUA.GNR, Pep@MUA.GNR-DOX, Apt-Pep@MUA.GNR-DOX, free DOX or PBS was investigated 30 days, post-administration.

Obtained results indicated that mice treated with either targeted or non-targeted peptosomes did not show considerable body weight changes during the experiment while mice receiving free DOX illustrated loss of body weight during the experiment as a result of free DOX systemic toxicity. In consistent with previous reports, free DOX exhibited severe systemic toxicity [82]. However, encapsulation of DOX in the stable vesicular structure of peptosomes significantly reduced its systemic toxicity in terms of survival percentage and alteration of body weight. In the current study, the targeted Apt-Pep@MUA.GNR-DOX demonstrated the best performance toward tumor growth suppression, loss of body weight and survival percentage.

### Biodistribution assessment using ex vivo florescence imaging

**Fig. 14:**
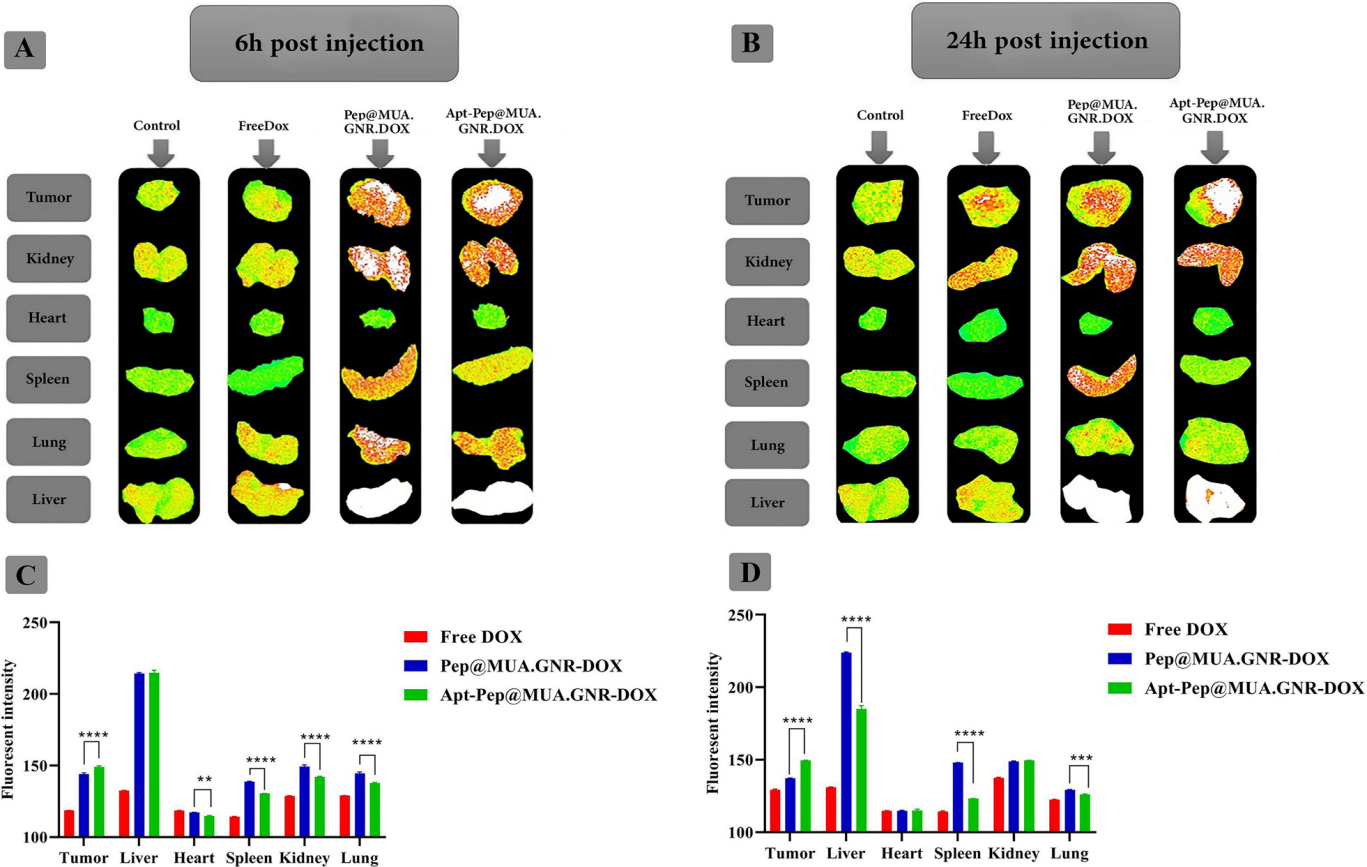
Ex vivo imaging of tumor tissues and mice organs. 6 h (**A**) and 24 h (**B**) after i.v injection of free DOX, Pep@MUA.GNR-DOX and Apt-Pep@MUA.GNR-DOX with equal DOX (5 mg/kg) and MUA.GNR (1 mg/kg) concentration. The Quantitative ROI analysis of DOX in tumor tissues and mice organs after 6 h (**C**) and 24 h (**D**) i.v. administration of either Apt-Pep@MUA.GNR-DOX, Pep@MUA.GNR-DOX or free DOX

The biodistribution of the formulations were evaluated after i.v. administration of free DOX, Pep@MUA.GNR-DOX and Apt-Pep@MUA.GNR-DOX (equivalent DOX concentration of 5 mg/kg) to the 4T1 tumorized BALB/c mice. In the next step, 6 and 24 h post-injection, mice were euthanized and major organs (kidney, spleen, liver, heart, and lung) were isolated and KODAK IS apparatus was used to prepare ex vivo fluorescence imaging using DOX fluorescence (Fig. 14).

A significant DOX accumulation in tumor tissues of mice receiving either Apt-Pep@MUA.GNR-DOX or Pep@MUA.GNR-DOX after 6 h i.v administration compared to those receiving free DOX were indicated which might be due to the longer blood circulation half-life and enhanced penetration into the tumor microenvironment via EPR effect. Enhanced tumor penetration of DOX loaded in targeted and non-targeted peptosomes improved the biodistribution of DOX after encapsulation in peptosome nanostructures. However, strongest DOX fluorescence intensity was shown in tumor tissue of mice after24 h injection of Apt-Pep@MUA.GNR-DOX (p ≤ 0.0001, n = 4). For mice receiving free DOX, fluorescence intensity in most organs and tumor tissue was very weak compared to those treated with either Apt-Pep@MUA.GNR-DOX or Pep@MUA.GNR-DOX, which is most likely due to the fast clearance of free DOX from the blood stream. On the other hands, DOX fluorescence intensity in organs of mice receiving Apt-Pep@MUA.GNR-DOX demonstrated higher tumor accumulation and lower major organs accumulation compared to those receiving Pep@MUA.GNR-DOX as a result of improved pharmacokinetics of the targeted peptosomes (Apt-Pep@MUA.GNR-DOX).

### In vivo CT scan imaging

Recent researches indicated that gold NPs have more advantages compared to FDA-approved iodinated contrast agents due to the high density and atomic number, desirable X-ray attenuation characteristics and adjustable shape, size and surface chemistry for special biomedical applications.

**Fig. 15:**
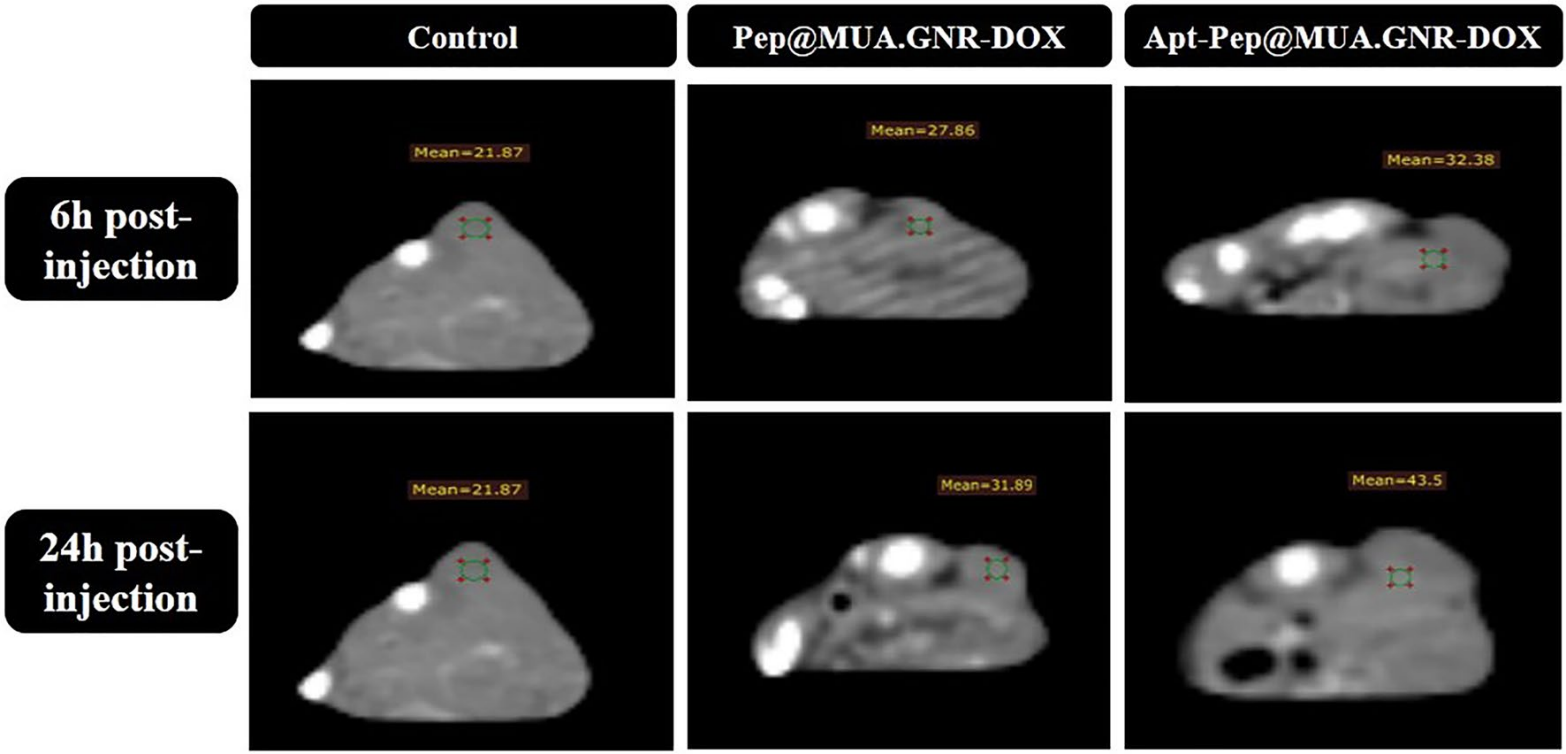
Clinical CT scan imaging of 4T1 tumor-bearing mice 6 and 24 h post-injection of either Pep@MUA.GNR-DOX or Apt-Pep@MUA.GNR-DOX

In the current study, Pep@MUA.GNR-DOX and Apt-Pep@MUA.GNR-DOX were employed as CT scan contrast agents due to encapsulation of the hydrophobic GNR in the bilayer of both argeted and non-targeted peptosomes. The diagnostic ability of the theranostic peptosomes was evaluated 6 and 24 h after i.v injection either Pep@MUA.GNR-DOX or Apt-Pep@MUA.GNR-DOX (150 µl of equivalent DOX concentration of 5 mg/kg, 1 mg/kg of MUA.GNR concentration to 4T1 tumorized BALB/c mice. According to CT scan imaging results (Fig. 15), the strongest CT signal intensity value was indicated in tumor tissue of mice receiving Apt-Pep@MUA.GNR-DOX compared to those treated with Pep@MUA.GNR-DOX. On the other hands, the animals treated with Pep@MUA.GNR-DOX indicated higher CT signal intensity values compared to that of control group (treated with 150 µl PBS) after 6 and 24 h of i.v administration.

**Fig. 16:**
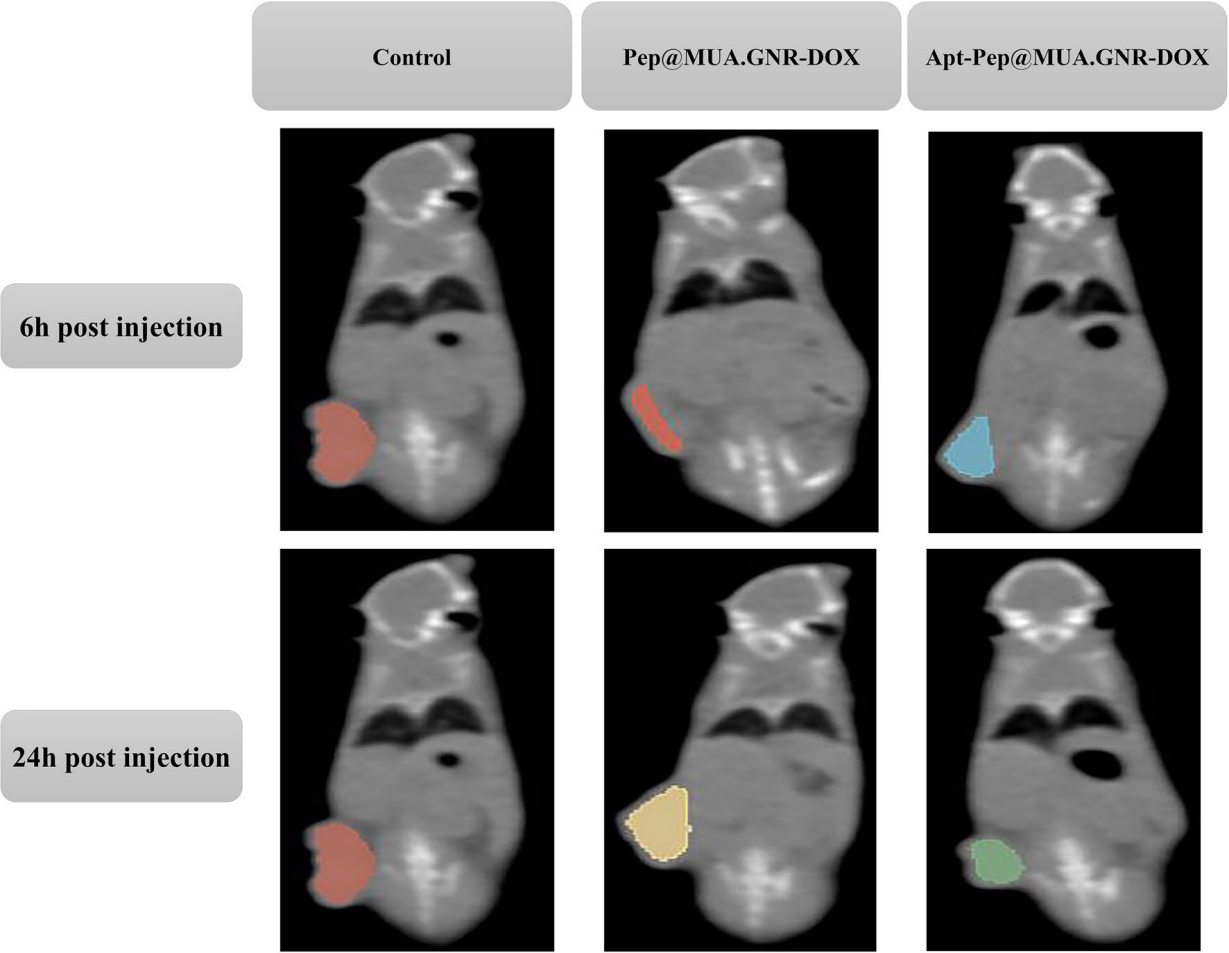
The in vivo CT scan coronal view of the tumor site 6 and 24 h post-injection of either Pep@MUA.GNR-DOX or Apt-Pep@MUA.GNR-DOX

In order to precisely investigate the theranostic efficiency of the prepared platforms, the in vivo CT scan coronal views of the mice tumors were prepared (Fig. 16) and the ROI of the tumors tissues were evaluated through 3D slicer (Version 4.11.20210226, https://www.slicer.org/) image segmentation software and CT signal intensity values were estimated in the whole tumor volume (Table 3). As represented in Table 3, the Hounsfield density in the tumor of mice treated with Apt-Pep@MUA.GNR-DOX was higher than those of treated with Pep@MUA.GNR-DOX 6 and 24 h post-injection. The obtained results demonstrated the capability and versatility of the prepared platform for in vivo CT imaging. The results of this study were clearly correlated with the data obtained from biodistribution investigation.

**Table 3:** Density (Hounsfield) of the 3D slicer image segmentation software in the coronal view CT scan images

Type of injected formulation	6 h post injection	24 h post injection
PBS	16.9	16.9
Pep@MUA.GNR-DOX	18	17.1
Apt-Pep@MUA.GNR-DOX	21.2	23.5

### Histopathological investigation

Pathological alterations of mice major organs were investigated 20 days after administration of free DOX, Pep@MUA.GNR, Pep@MUA.GNR-DOX and Apt-Pep@MUA.GNR-DOX with same DOX (5 mg/kg) and MUA.GNR (1 mg/kg) concentration.

**Fig. 17:**
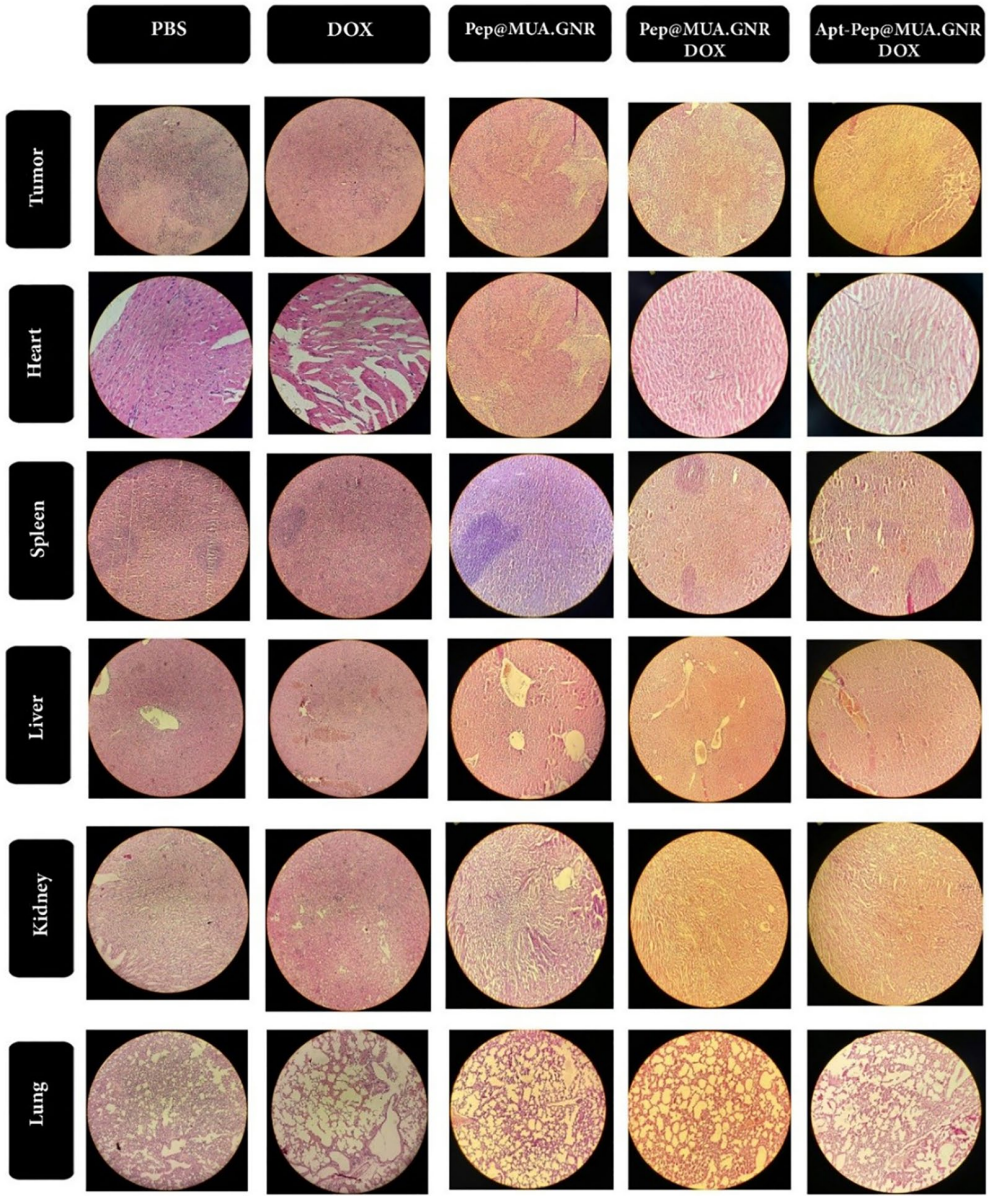
Hematoxylin and eosin staining of tumor tissue and mice organs in 4T1 tumoric mice, 20 days i.v. injection of either PBS, free DOX, Pep@MUA.GNR, Pep@MUA.GNR-DOX or Apt-Pep@MUA.GNR-DOX with equal concentration of DOX (5 mg/kg) and MUA.GNR (1 mg/kg)

According to H&E staining of tumors sections (Figs.16, 17), the tumor specimen of mice treated with either Pep@MUA.GNR-DOX or Apt-Pep@MUA.GNR-DOX showed greater necrotic area compared to those treated with either free DOX or PBS. Furthermore, the necrotic regions in mice treated with Apt-Pep@MUA.GNR-DOX was wider compared to all other treatment groups, which could be ascribed to the strong tumor accumulation capability of the targeted system. The cardiotoxicity in mice treated with free DOX was demonstrated through intense pathological atrophy of heart tissue that is one of the major side effect of free DOX [7, 81, 84]. The prepared peptosomal formulation showed no obvious cardiotoxicity in terms of pathological deformations (Fig. 17).

## Materials and methods

### Materials

HAuCl_4_, AgNO_3_, NaBH_4_, CTAB, ascorbic acid, MUA**,** *n*-hexylamine, EDC, NHS and 3-(4,5-dimethylthiazol-2-yl)-2,5-diphenyltetrazolium bromide (MTT) were obtained from Sigma‐Aldrich Co. (St. Louis, MO). Hetero-bifunctional polyethylene glycol (PEG) with maleamide and carboxylic acid as end terminal group with Mw of 5 kDa which was bought from (Mal-PEG- JenKem Technology USA Inc. (Beijing, China). BLG-NCA was procured from Hangzhou Yuhao Chemical TechnologyCo. Ltd. DOX was bought from Euroasia Co., Ltd., India. All solvents utilized in this report were bought from Merck, Darmstadt, Germany without further purification.

Trypsin, penicillin–streptomycin, Roswell Park Memorial Institute (RPMI) 1640 medium, FBS were obtained from GIBCO, Darmstadt, Germany. MCF-7 (human breast cancer cell line), 4T1 (mice metastatic breast cancer) and CHO (Chinese hamster ovary) cell lines were bought from Pasteur Institute of Iran. All cell lines were maintained in RPMI-1640 medium containing 10% of FBS and 1% streptomycin-penicillin (100 μg/ml streptomycin and 100 units/ml of penicillin) in an incubator at 37 °C under 5% CO_2_ atmosphere and 95% humidity.

### Synthesis of polymers

#### Ring-Opening polymerization of BLG-NCA

PBLG was prepared through ROP of BLG-NCA monomer in dry DMF using *n*-hexylamine as an initiator and the molar ratio of BLG-NCA (M) to n-hexylamine (I) was adjusted 120 (M/I) [46]. Firstly, BLG-NCA (3 mmol, 0.789 g) was dissolved in dry DMF (7.894 ml) in a two necked flask, 50 ml under an argon atmosphere, then *n*-hexylamine solution (0.025 mmol, 3.3 µl) in dry DMF (189 µl) was added dropwise to the BLG-NCA solution and was stirred at 25 °C for 3 days. Then polymer was purified through precipitation with adding excessive diethyl ether (20 ml) for tree times to produce a white solid PBLG. Then, the purified polymer was dried under vacuum at room temperature for 24 h.

#### Synthesis of polyethylene glycol-*block*-poly(γ-benzyl L-glutamate) (PEG-PBLG)

The carboxylic acid of Mal-PEG-COOH was covalently coupled to the terminal primary amines of PBLG through EDC/NHS chemistry. In this regard, 0.02 mM (0.1 g), 5 mg of Mal-PEG-COOH (5 kDa MW) was dissolved in 5 ml of dry DMF. Then, EDC (PEG:EDC molar ratio of 1:8) and NHS (PEG:NHS molar ratio of 1:8) were added to the above solution and stirred for 2 h to activate the carboxylic acid group of Mal-PEG-COOH at room temperature. In the next step, addition of 0.02 mM of PBLG (0.29 g) was performed to the prior solution and stirred overnight. The copolymer was precipitated in 15 ml cold diethyl ether to obtain a white sediment of block copolymer and washed 3 times with 15 ml methanol:diethyl ether solution (30:70) to remove unreacted EDC and NHS. The final PEG-PBLG block copolymer was freeze dried and stored at 20 °C until use [7].

#### Polymer characterization

The ^1^H-NMR spectra of PBLG and Mal-PEG-PBLG diblock at room temperature in CDCl_3_ and DMSO-d_6_ respectively and CNMR spectra of Mal-PEG-COOH, PBLG and Mal-PEG-PBLG diblock at room temperature in DMSO-d_6**,**_ CDCl_3_ and DMSO-d_6_ respectively were recorded using Bruker Avance 300 MHz NMR spectrometer. Moreover, the chemical structures of PBLG and Mal-PEG-PBLG diblock copolymer were assessed via FTIR analysis using Perkin-Elmer Model 1000. Molecular weight and polydispersity of PBLG polymer was determined using GPC system (Shimadzu LC-20A) with StyragelHR2 Column model in THF as solvent with a flow rate of 1.0 ml/min at 35 °C.

The thermal properties of Mal-PEG-COOH, PBLG and Mal-PEG-PBLG diblock copolymer were evaluated through DSC (Mettler Toledo DSC 822, Greifensee, Switzerland). For DSC, 2 mg of block copolymer was analyzed in thermal cycles from 0 to 200 °C with heating rate of 10 °C/min. The thermal stability of the Mal-PEG-COOH, PBLG and Mal-PEG-PBLG were investigated via thermo-gravimetric analysis (TGA) under nitrogen atmosphere at heating rate of 10 °C/min from room temperatures to 600 °C.

#### Synthesis of GNR

The GNR was prepared via seedless growth method according to the previous report [49]. In this regard, CTAB (0.2 M, 1.092 g) was poured in round-bottom flask, 100 ml and dissolved in 15 ml of deionized water. After obtaining the clear CTAB solution, an aqueous solution containing HAuCl_4_ (15 ml, 1 mM) was added, after 10 min, AgNO_3_ (900 µL, 4.0 mM), HCl (36 µL, 37%) and ascorbic acid (225 µL, 85.8 mM) were added respectively and gently stirred until colorless. Immediately, the freshly prepared ice-cold aqueous solution of NaBH_4_ (22.5 µL, 0.1 M) was added to the reaction mixture and kept overnight without stirring at constant temperature (30 °C). The appearance of dark red color demonstrated the successfully synthesized of GNR. The synthesized GNRs were centrifuged (15,000 rpm, 20 min) and elimination of residual CTAB was resulted through 3 times washing with deionized water (15 ml). Afterward the final product was dispersed in 5 ml of deionized water.

#### Ligand exchange and synthesis of hydrophobic GNR

Hydrophobic GNR was fabricated through ligand exchange method. Briefly, ethanol solution of MDA (0.02 mM, 1 mL) was added dropwise to GNRs aqueous solution (20 nM, 5 mL) under mild stirring. Then solution was stirred overnight at ambient temperature. Afterwards, excess ligand was eliminated via chloroform (500 µl) extraction for three rounds and the GNRs were collected by centrifugation (15,000 rpm, 20 min) and suspended in 1 mL of THF.

#### Loading of DOX and MUA.GNR (Pep@MUA.GNR-DOX)

Peptosomes co-loaded with DOX/GNR were prepared through double emulsion method. In the first step, PEG-PBLG (5 mg) was dissolved in dichloromethane (800 µL) and 200 µL of MUA.GNR was mixed to the solution. Afterwards, DOX aqueous solution (2 mg/mL, 100 µL) was added dropwise to the solution under probe-sonication for 5 min to form emulsified solution (named E1). Then, the dropwise addition of E1 was performed to 4 mL of PVA 0.5% in PBS during sonication for 15 min (named E2). In the next step, the dropwise addition of E2 to 10 ml PVA 0.1% in PBS was performed under stirring and the colloidal suspension was further stirred overnight at 800 rpm. In the final step, elimination of free DOX and residual PVA were carried out via washing of Pep@MUA.GNR-DOXwith 2 ml of deionized water for 2 times through centrifugation at 15,000 g for 20 min.

The amount of encapsulated DOX was indirectly determined by calculating the unloaded DOX in supernatant by UV spectrophotometer at 480 nm. Then LC and encapsulation EE of DOX in peptosomes were estimated according to the following formulas, respectively.

$${\text{LC }}\left( \% \right) \, = \left( {\frac{{\text{Amount of DOX loaded in formulation}}}{{{\text{Total amount of formulation}} }}} \right) \times { 1}00$$

$${\text{EE }}\left( \% \right) \, = \, \left( {\frac{{\text{Amount of DOX loaded in formulation}}}{{\text{Total amount of DOX added in formulation}}}} \right) \, \times { 1}00$$

Hydrophobic MUA.GNR was encapsulated in peptsomes via single emulsion method. In the first step, PEG-PBLG (5 mg) was dissolved in dichloromethane (800 µL) and 200 µL of MUA.GNR was mixed with the solution. Then, the dropwise addition of mixture was done to 4 mL of PVA 0.5% in PBS with sonication for 15 min. In the next step, the slowly addition of emulsified solution to stirring PVA solution (0.1% in PBS, 10 ml) was performed under stirring condition and the colloidal suspension was further stirred overnight at 800 rpm. Finally, Pep@MUA.GNR was centrifuged at 15,000 g for 20 min and washed twice with 2 ml deionized water twice to remove the PVA.

The loading content of gold NRs in the formulations was measured via ICP-OES. In addition, morphology and size of MUA-GNR were investigated with TEM (a Leo 912AB microscope (Germany) operated at 120 kV).

### DOX release profiles of Pep@MUA.GNR-DOX

The release profiles of DOX were studied in 3 different release media including PBS pH 7.4, PBS supplemented with 30% FBS and citrate buffer pH 5.4. For this study, 1.5 mL of the suspended Pep@MUA.GNR-DOX (with 500 µg/ml DOX content) in release medium was poured into a dialysis sac (MW cut off = 6–8 kDa) which immersed in 20 mL buffer (citrate or PBS), then was shaked on an incubator at 80 rpm and 37 °C. One mL of sample was withdrawn at various time points over the 10 days (1, 2, 3, 4, 5, 24, 48, 72, 96, 120, 144, 168, 192, 216, 240 h) and each sample was replaced with 1 ml of fresh media (PBS or citrate buffer). The fluorescence spectroscopy was utilized for DOX content of the collected samples and excitation and emission wavelength were adjusted at 480 and 580 nm respectively.

### In vitro colloidal stability of peptosomes

The serum stability of targeted and non-targeted peptosomes were investigated in PBS media supplemented with 10% for 48 h. In this study, 1 ml of prepared peptosomes (Apt-Pep@MUA-GNR.DOX and Pep@MUA-GNR.DOX) were dispersed in PBS including 10% FBS and located within a shaker incubator at 37 °C. Afterward, size and polydispersity of nanopeptosomes were analyzed using DLS method over the period of 48 h. Furthermore, the shelf life of Apt-Pep@MUA-GNR.DOX and Pep@MUA-GNR.DOX were investigated after 30 days stored at 4 °C.

### Conjugation of EpCAM aptamer to Pep@MUA.GNR-DOX surface

Thiolated EpCAM aptamer [42, 43] was coupled to the surface of Mal-PEG-PBLG peptosomes through thiol-maleimide reaction. EpCAM aptamer (10 µm, 20 µl) was added to the aqueous suspension of Pep@MUA.GNR-DOX nanoformulation 5 mg/ml in nuclease-free water and stirred at 4° C overnight. Finally, the unreacted aptamers was removed by centrifugation (30 min, 15,000 rpm). The free non-conjugated aptamer in supernatant was analyzed with UV spectroscopy (absorbance at 260 nm) to indirectly calculate the amount of conjugated aptamer.

### Physiochemical properties of GNR and Pep@MUA.GNR-DOX nanoformulation

The suspensions of NPs in deionized water were prepared (0.5 mg/mL) and their average size, polydispersity index and zeta potential were analyzed implementing Dynamic Light Scattering (DLS) method by a Nanopartica SZ-100; HORIBA Ltd, Kyoto, Japan at 25 °C.

Field emission scanning electron microscope (FESEM, TESCAN BRNO- Mira3 LMU, Czech Republic) and atomic force microscope (AFM, JPK Nano-Wizard II, Germany) were utilized to investigate the particle size, polydispersity and morphology of the Pep@MUA.GNR-DOX and Apt-Pep@MUA.GNR-DOX systems. The samples for FESEM imaging were diluted in deionized water (0.5 mg/mL) and dropped onto metal stub. Afterwards, the samples were dried and a gold film (200 A^o^) was covered on their surface under vacuum. An accelerating voltage (10 kV) was applied for SEM imaging.

For AFM imaging, the samples were diluted in deionized water (0.5 mg/mL) and dried onto the mica disc at room temperature. Imaging was performed in non-contact mode and dehydrated state. Optical properties of GNR and MUA-GNR were analyzed using an CARY 100 UV/Vis spectra (Varian).

### In vitro cytotoxicity evaluation

The in vitro cytotoxicity of free DOX, targeted and non-targeted systems with equal concentrations of DOX on cancer cells (4T1 and MCF-7) and normal cells (CHO) were investigated. For this purpose, MCF-7, CHO and 4T1 cell lines were grown in RPMI medium, supplemented with 1% penicillin–streptomycin and 10% (v/v) fetal bovine serum (FBS) in incubator under 5% CO_2_ and 95% humidity at 37 °C. Each well of 96-well plates were seeded with cell lines at a density of 5 × 10^3^ with 100 μL of culture media and incubated at 37 °C overnight. Then, different cell lines were treated with the fresh culture media containing Pep@DOX, Pep@MUA.GNR, Pep@MUA.GNR-DOX, Apt-Pep@MUA.GNR-DOX and free DOX (equivalent concentrations of DOX from 0.3125 to 20 µg/ml with 4 replicate for each concentration, equivalent concentration of carrier for drug free systems) for 6 h [62]. Then, culture media were replaced with 100 µl of fresh RPMI medium and further incubated at 37 °C for 48 h. Thereafter, 20 µl of MTT solution in PBS (5 mg/mL) was poured to each well and placed at incubator for 4 h. Finally, the media was eliminated and DMSO (100 µl) was poured to each well and absorbance was measured at 570 nm with reference wavelength of 630 nm using microplate reader (Tecan Group Ltd., Switzerland).

### DOX cellular internalization analysis through flowcytometry

The cellular uptake of Pep@MUA.GNR-DOX and Apt-Pep@MUA.GNR-DOX was investigated through BD FACS Calibur equipped with 488 lasers in the FL2 channel. In this study, 4T1 or CHO cell lines (5 × 10^4^) were seeded in each well of 24-well plates and incubated overnight. Afterwards, the cell lines were exposed to either free DOX, Pep@MUA.GNR-DOX or Apt-Pep@MUA.GNR-DOX with same concentration of DOX (5 µg/mL,600 µL) for 2 h. In the next step, RPMI media were removed, and then the cells were washed 2 times with cold PBS, trypsinized and centrifuged at 1400 rpm for 7 min. Afterwards, the supernatant was removed and cell pallets were washed with cold PBS pH 7.4 for three times (1400 rpm for 7 min). Thereafter, the cell pallets were resuspended in 200 µL of cold PBS for flow cytometry analysis. Finally, flowJo 7.6 software was utilized for data analysis [29].

### In vivo antitumor efficacy

All animal experiments were performed with the approval of both the Institutional Ethical Committee and Research Advisory Committee of the School of Pharmacy, Mashhad University of Medical Sciences. Female BALB/c mouse (4 to 6 weeks old) were purchased from Pasteur Institute of Iran (Tehran, Iran) and applied for in vivo experiments*.* The right flanks of the mice were subcutaneously implanted with 4T1 cell suspension (80 µL of 4 × 10^5^ cells in PBS). One week after tumor cells implantation, the mice were divided into 5 groups with 5 mice in each group. The study was started when the tumor volume received 20–30 mm^3^. In this regard, 150 µl of either free DOX, Pep@MUA.GNR, Pep@MUA.GNR-DOX, or Apt-Pep@MUA.GNR-DOX with same DOX and MUA.GNR concentration (5 mg/kg and 1 mg/kg, respectively) and 297 mg/kg of free drug carrier were intravenously injected. In the current study, PBS was utilized as negative control. Tumor volume and weight of each mouse were measured for 30 days post injection. For assessment of tumor size, the following equation was applied [58].

$${\text{Tumor volume}}\, = \,\frac{{{\text{smallest diameters of tumor }} \times {\text{largest diameters of tumor }} \times {\text{ depth of tumor}}}}{2}$$

Monitoring of the body weight and the survival rates of mice were followed during the experiment for demonstration of systemic toxicity. It should be noted that the mice of each group with the tumor volume more than 1.5 cm^3^ or weight loss more than 20% was euthanized [7].

### Ex vivo biodistribution

The biodistribution assay of DOX in targeted and non-targeted formulations compared to free DOX and negative control were evaluated using ex vivo fluorescence imaging. Briefly, a suspension of 4T1 cells in PBS (4 × 10^5^ cells in 80 µL) was subcutaneously implanted in Female BALB/c mice. When the tumor volume reached 200 mm^3^, mice were divided into 3 groups and 150 μL of either free DOX, Pep@MUA.GNR-DOX orApt-Pep@MUA.GNR-DOX with equal concentration of DOX (5 mg/kg) were i.v. administrated to tail vein. Afterwards, the mice were euthanized 6 and 24 h after i.v. administration, tumor, spleen, liver, heart, kidneys and lungs were isolated and ex vivo imaging was done implementing KODAK IS in vivo imaging system in excitation and emission wavelengths of 480 and 580 nm, respectively. Furthermore, KODAK Molecular Imaging® software 5.0 was applied for quantitatively determination of the fluorescence intensity of DOX in various organs [58].

### In vivo CT scan imaging

Clinical CT scan imaging of Female BALB/c mice tumorized with 4T1 cells (with 200 mm^3^ tumor volume) was performed after 6 and 24 h i.v. injection of the Pep@MUA.GNR-DOX, Apt-Pep@MUA.GNR-DOX(150 µL and 5 mg/kg equal concentration of DOX, 1 mg/kg MUA-GNR) using clinical CT scanner (Somatom Volume Zoom; Siemens Medical Systems, Erlangen, Germany). After administration of the systems (6 h and 24 h post-administration), the tumorized mice were anesthetized and fixed on a holder while locating on the drill chuck of the rotational axis of the CT Scan. For CT imaging, the parameters were adjusted in tube voltage of 120 kV, 150 mAs, slice thickness of 0.2 cm, rotation time 0.75 s and feed rotation of 0.5 mm [85]. Moreover, the in vivo CT scan coronal views of the tumors were prepared and analyzed by the 3D slicer (Version 4.11.20210226, https://www.slicer.org/) image segmentation for determining the ROI of tumors tissues.

### Histopathological investigation

Twenty days post-intravenous injection of either 150 µL of free DOX, Pep@MUA.GNR-DOX, Apt-Pep@MUA.GNR-DOX or Pep@MUA.GNR with equal DOX and MUA-GNR concentration (5 mg/kg and 1 mg/kg), one mouse in each group was euthanized. Afterwards, the important organs of mouse in each group comprising tumor, spleen, liver, heart, kidneys and lungs were separated and fixed in 10% formalin solution after 3 times PBS washing. Next, standard rotary microtome was utilized to prepare the tissue sections with 4–5 μm thickness and were stained with hematoxylin and eosin (H &E). The optical microscope at 10X magnification was utilized for imaging [29].

### Statistics

Statistical data analysis was performed through two-way ANOVA (analysis of variance). Difference between the mean of data were statistically significant through a probability value ≤ 0.05. Additionally, the results were reported as mean ± standard error of standard deviation (SD).

## Conclusion

In this study, for the first time, a targeted theranostic peptosomes based on polypeptide- amphiphilic diblock copolymer of PEG-PBLG was developed. In this system, DOX and hydrophobic GNR (MUA.GNR) were encapsulated in aqueous core and hydrophobic bilayer of peptosomes, respectively through double emulsion method to produced Pep@MUA.GNR-DOX.

The peptosomes surface was tagged with the thiol-modified DNA aptamer of EpCAM through the maleamide functionality present at the end of the PEG block, exposed on their surface (Apt-Pep@MUA.GNR-DOX). In vitro assessments showed higher cellular toxicity and cellular internalization of Apt-Pep@MUA.GNR-DOX in EpCAM overexpressing breast cancer cell lines (4T1 and MCF-7) in comparison with that of Pep@MUA.GNR-DOX. Furthermore, the results of release profile in PBS buffer supplemented with 30% FBS and stability test in the presence of 10% FBS demonstrated considerable stability of the prepared peptosomes in biological conditions.

The preclinical data in 4T1-tumorized mice indicated higher tumor accumulation, lower systemic toxicity and considerable therapeutic index of the Apt-Pep@MUA.GNR-DOX while showing its versatility toward in vivo CT imaging.

The important features of the prepared platform included (1) biocompatibility and vesicular structure of the vehicle; (2) co-delivery of anticancer hydrophilic drug, doxorubicin and hydrophobic gold nanorods as biocompatible, versatile contrast agent (3) decorating the DNA aptamer against EpCAM on their surface for providing guided drug delivery. The obtained data showed the prepared platform has efficient tumor accumulation, ideal therapeutic index, acceptable safety profile while providing acceptable contrast in tumor tissue even 24 h post-administration.

Thus, the prepared innovative targeted theranostic peptosomes based on PEG-PBLG amphiphilic block copolymer which encapsulated gold nanorods could serve as an operative multimodal platform for the treatment and imaging, allowing oncologist to monitor real-time response of the patients to chemotherapy.

## Data Availability

All data during the current report are available by corresponding authors upon reasonable request.
